# Molecular mechanism of Danshenol C in reversing peritoneal fibrosis: novel network pharmacological analysis and biological validation

**DOI:** 10.1186/s12906-023-04170-x

**Published:** 2023-10-13

**Authors:** Jiabin Liang, Lulu Cheng, Jie Feng, Zeping Han, Chen Huang, Fangmei Xie, Yongsheng Li, Xun Luo, Qingmei Wang, Jinhua He, Hanwei Chen

**Affiliations:** 1 Guangzhou Panyu Central Hospital, Guangzhou University of Chinese Medicine, Guangzhou, China; 2 Radiology Department of Sun Yat-Sen Memorial Hospital, Sun Yat-Sen University, Guangzhou, China; 3 Medical Imaging Institute of Panyu, Guangzhou, China; 4 Guangzhou Municipality Tianhe Nuoya Bio-Engineering Co., Ltd, Guangzhou, China; 5 Kerry Rehabilitation Medicine Research Institute, Shenzhen, China; 6 Stroke Biological Recovery Laboratory, Teaching Affiliate of Harvard Medical School, Spaulding Rehabilitation Hospital, Charlestown, MA USA; 7 Panyu Health Management Center (Panyu Rehabilitation Hospital), Guangzhou, China

**Keywords:** Danshenol C, Network pharmacology, Biological validation, Peritoneal fibrosis, Molecular mechanism

## Abstract

**Objective:**

The primary objective of this study is to elucidate the molecular mechanism underlying the reversal of peritoneal fibrosis (PF) by Danshenol C, a natural compound derived from the traditional Chinese medicine Salvia miltiorrhiza. By comprehensively investigating the intricate interactions and signaling pathways involved in Danshenol C's therapeutic effects on PF, we aim to unveil novel insights into its pharmacological actions. This investigation holds the potential to revolutionize the clinical application of Salvia miltiorrhiza in traditional Chinese medicine, offering promising new avenues for the treatment of PF and paving the way for evidence-based therapeutic interventions.

**Methods:**

Firstly, we utilized the YaTCM database to retrieve the structural formula of Danshenol C, while the SwissTargetPrediction platform facilitated the prediction of its potential drug targets. To gain insights into the genetic basis of PF, we acquired the GSE92453 dataset and GPL6480-9577 expression profile from the GEO database, followed by obtaining disease-related genes of PF from major disease databases. R software was then employed to screen for DEG associated with PF. To explore the intricate interactions between Danshenol C's active component targets, we utilized the String database and Cytoscape3.7.2 software to construct a PPI network. Further analysis in Cytoscape3.7.2 enabled the identification of core modules within the PPI network, elucidating key targets and molecular pathways critical to Danshenol C's therapeutic actions. Subsequently, we employed R to perform GO and KEGG pathway enrichment analyses, providing valuable insights into the functional implications and potential biological mechanisms of Danshenol C in the context of PF. To investigate the binding interactions between the core active components and key targets, we conducted docking studies using Chem3D, autoDock1.5.6, SYBYL2.0, and PYMOL2.4 software. We applied in vivo and in vitro experiments to prove that Danshenol C can improve PF. In order to verify the potential gene and molecular mechanism of Danshenol C to reverse PF, we used quantitative PCR, western blot, and apoptosis, ensuring robust and reliable verification of the results.

**Results:**

① Wogonin, sitosterol, and Signal Transducer and Activator of Transcription 5 (STAT5) emerged as the most significant constituents among the small-molecule active compounds and gene targets investigated. ②38 targets intersected with the disease, among which MAPK14, CASP3, MAPK8 and STAT3 may be the key targets; The results of GO and KEGG analysis showed that there was a correlation between inflammatory pathway and Apoptosis. ④Real-time PCR showed that the mRNA expressions of MAPK8 (JNK1), MAPK14 (P38) and STAT3 were significantly decreased after Danshenol C treatment (*P* < 0.05), while the mRNA expression of CASP3 was significantly increased (*P* < 0.05)⑤Western blot showed that protein expressions of CASP3 and MAPK14 were significantly increased (*P* < 0.05), while the expression of STAT3 and MAPK8 was decreased after Danshenol C treatment (*P* < 0.05). ⑥There was no significant difference in flow analysis of apoptosis among groups.

**Conclusion:**

The findings suggest that Danshenol C may modulate crucial molecular pathways, including the MAPK, Apoptosis, Calcium signaling, JAK-STAT signaling, and TNF signaling pathways. This regulation is mediated through the modulation of core targets such as STAT3, MAPK14, MAPK8, CASP3, and others. By targeting these key molecular players, Danshenol C exhibits the potential to regulate cellular responses to chemical stress and inflammatory stimuli. The identification of these molecular targets and pathways represents a significant step forward in understanding the molecular basis of Danshenol C's therapeutic effects in PF. This preliminary exploration provides novel avenues for the development of anti-PF treatment strategies and the discovery of potential therapeutic agents. By targeting specific core targets and pathways, Danshenol C opens up new possibilities for the development of more effective and targeted drugs to combat PF. These findings have the potential to transform the landscape of PF treatment and offer valuable insights for future research and drug development endeavors.

**Supplementary Information:**

The online version contains supplementary material available at 10.1186/s12906-023-04170-x.

## Background

Peritoneal dialysis (PD) serves as a vital renal replacement therapy for patients with end-stage renal disease (ESRD), involving the exchange of water and solutes between the dialysate and intraperitoneal capillaries through the peritoneum. Globally, approximately 11% of the dialysis population comprises PD patients, highlighting its significance in ESRD management [1, 2]. Notably, PD stands as the preferred approach for integrated treatment, boasting advantages such as conservation of medical resources, straightforward implementation, minimal impact on hemodynamics, better preservation of residual renal function, and improved quality of life for patients [3]. However, PF emerges as a major concern in PD patients, driving structural changes in the peritoneum and resulting in ultrafiltration failure (UFF). PF represents a critical factor leading to patient withdrawal from PD treatment [4]. Characterized by abnormal production of extracellular matrix proteins, PF causes damage to peritoneal mesothelial cells, thickening of connective tissue in the peritoneal interstitial layer, and neovascularization, making it one of the prominent and serious complications of PD [5]. Although the exact pathogenesis of PF remains incompletely understood, modern medicine primarily relies on symptomatic treatments, such as enhancing the biocompatibility of peritoneal dialysis solutions (PDS) and employing renin-angiotensin system blocking agents [6]. However, these approaches offer limited prevention and treatment strategies. In recent years, traditional Chinese medicine (TCM) has emerged as a promising avenue for the prevention and treatment of PF associated with PD. Notably, TCM interventions have gained traction due to their notable safety profile and relatively cost-effective nature, gradually becoming a mainstream approach in managing PF. This article highlights the significance of exploring TCM-based preventive and therapeutic modalities to address PF in PD patients. As TCM interventions offer potential benefits and represent a hot area of research, their integration into PD treatment holds promise for advancing the management of PF [7, 8].

**Fig. 1:**
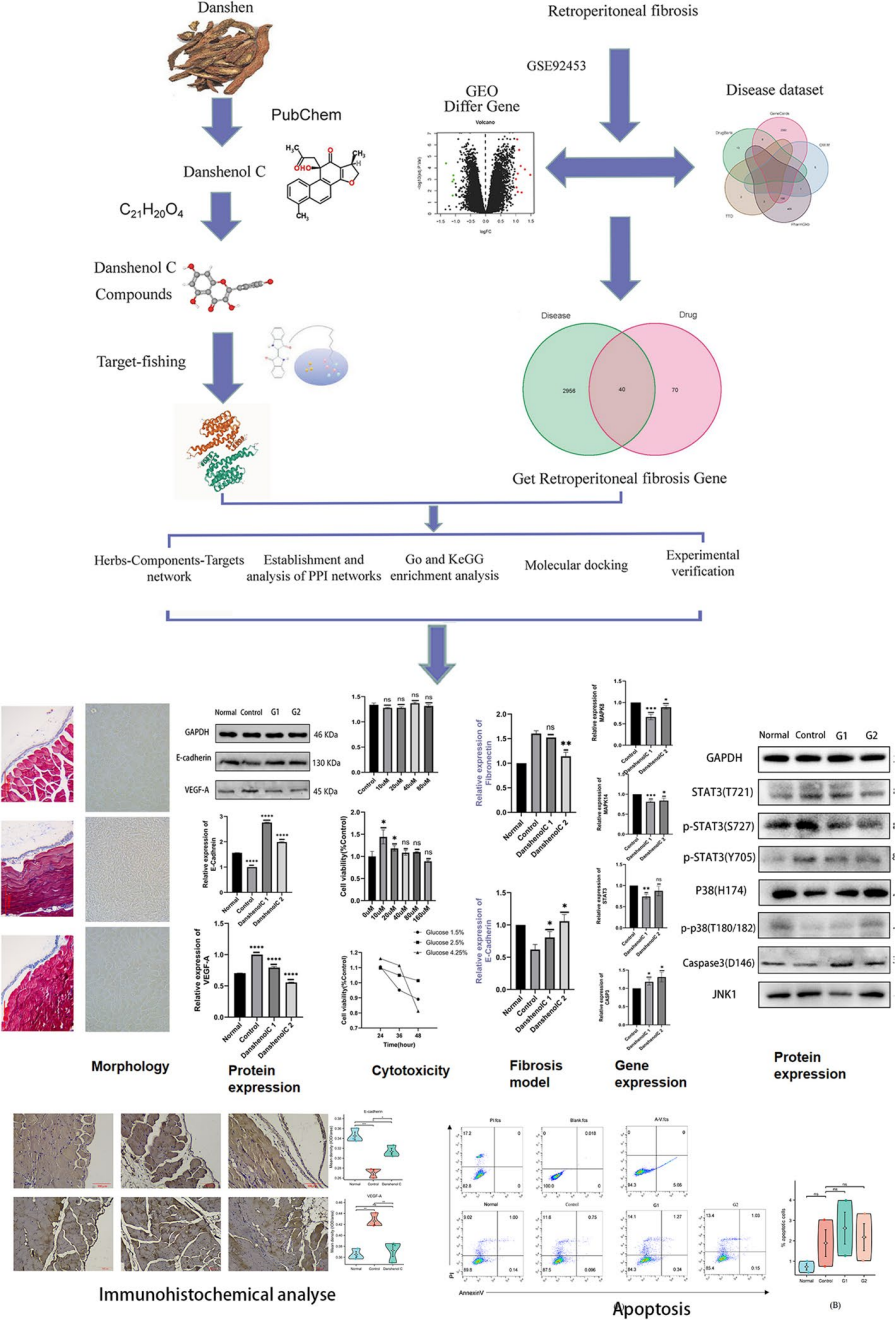
Flow chart

Salvia miltiorrhiza Bge, a medicinal plant belonging to the Labiaceae family, has been traditionally utilized for its therapeutic properties, primarily sourced from its dried roots and rhizomes. In recent times, modern research has revealed the presence of key chemical constituents in Salvia miltiorrhiza Bge, such as salvianolic acid B, cryptotanshinone, tanshinone IIA, protocatechualdehyde, and others. These constituents have demonstrated noteworthy pharmacological effects, encompassing anti-myocardial ischemia, anti-tumor, and anti-inflammatory properties [9, 10]. Notably, tanshinone IIA has been identified as a potent inhibitor of peritoneal mesothelial cell proliferation triggered by high glucose, thereby impeding PF formation [11]. Among the water-soluble natural compounds derived from Salvia miltiorrhiza Bge is Danshenol, which comprises Danshenol A, Danshenol B, and the recently discovered Danshenol C. The molecular formula of Danshenol C is represented as C21H20O4, and its structure is depicted in Fig. 1. The present study, for the first time, investigates the potential of Danshenol C as a novel therapeutic compound for PF treatment. This pioneering investigation lays a robust scientific foundation for future endeavors concerning the clinical development of related drugs, opening avenues for targeted and effective therapeutic strategies in the realm of PF management.

In this study, we employed cutting-edge network pharmacology technology to explore the intricate interactions between drugs and their targets. Through comprehensive searches across gene, protein, disease, and drug databases, coupled with real experimental data, we constructed a multi-dimensional relationship network encompassing "drug-gene-target-disease" interactions. By integrating these diverse data sources, we aimed to gain a comprehensive understanding of the complex biological network and investigate how drugs can potentially restore its balance. To shed light on the therapeutic efficacy and underlying molecular mechanisms of Danshenol C at different concentrations, we conducted in vitro experiments on human peritoneal mesothelial cells (HmRSV5) treated with high glucose peritoneal dialysate. The exploration of Danshenol C's effects in this context holds significant implications for understanding its potential therapeutic applications.

Through this integrative approach, we seek to unveil the therapeutic potential of Danshenol C and its capacity to modulate critical molecular pathways in the context of high glucose peritoneal dialysate treatment. The insights gained from this investigation will not only contribute to future basic research endeavors but also hold promising implications for potential clinical applications. By elucidating the intricate interactions between drugs, targets, and diseases, our study aims to provide a valuable reference for the development of targeted therapies and further advancements in the field of network pharmacology. Additionally, our findings may serve as a basis for future clinical applications of Danshenol C in treating peritoneal disorders, thus bolstering the arsenal of effective therapeutic interventions in medical practice (Fig. 1).

## Novel network pharmacological analysis

### Active ingredients and target screening of Danshenol C

Danshenol C, a bioactive compound derived from Danshen (Salvia miltiorrhiza), exhibits promising pharmacological properties, making it a potential candidate for therapeutic applications. However, despite its potential, the identification of specific drug targets for Danshenol C remains elusive within commonly used databases such as TCMSP, TCMID, ETCM, and others. To address this gap, we conducted an innovative network pharmacological analysis by exploring the YaTCM database (http://cadd.pharmacy.nankai.edu.cn/yatcm/help) for comprehensive information on Danshenol C [12]. Subsequently, the SwissTargetPrediction database [13] was utilized to predict potential targets of the active components, and to ensure accuracy and reliability, the collected targets were subjected to normalization with reference to the protein database Uniprot (https://www.uniprot.org/) using R4.1.0.

### Screening of Danshenol C differentially expressed genes

To address this knowledge gap, we conducted a comprehensive network pharmacological analysis. Initially, relevant datasets related to PF were meticulously screened in the GEO DataSets database, using "peritoneal fibrosis" as the primary keyword. Subsequently, we performed data processing and rigorous statistical analysis utilizing the Bioconductor package of R software, ensuring the robustness and reliability of our results.

To enable standardized gene representation for further analysis, we harnessed the Perl language to convert the probe-level expression data into gene symbol level data. This transformation facilitated a more accessible and consistent representation of gene information, critical for subsequent investigations. To identify DEGs between peritoneal fibrotic tissues and normal human tissues, we employed the Limma package of R language, a powerful tool renowned for its utility in microarray data analysis. This approach enabled the identification of gene expression patterns associated with the pathological condition, providing valuable insights into the molecular landscape of PF.

To comprehend the molecular targets implicated in PF, we curated pertinent information from authoritative databases, including GeneCards, PharmGKB, DrugBank, OMIM, and TDD. These comprehensive resources furnish a wealth of knowledge concerning disease-related genes and their functional implications, augmenting the scope and accuracy of our investigation.By intersecting the PF targets obtained from each database with the known targets of Danshenol C, we successfully identified common targets that mediate Danshenol C's beneficial effects in PF.

### Construction of active ingredient-PF target network

The exploration of potential drug targets for Danshenol C and the identification of DEG targets in PF were carried out employing a methodical approach. R software was utilized for data visualization, wherein the potential drug targets of Danshenol C and the DEG targets of PF were depicted. To ensure accuracy and consistency in target site nomenclature, Perl software was employed to correct the names of target sites. Furthermore, the Uniprot database [14] was referenced to select targets specific to the species "Homo sapiens." Subsequently, to construct the PPI network, the STRING database was accessed, with a confidence threshold of 0.4 employed to filter interactions.

To enhance clarity and focus on relevant interactions, disconnected nodes in the network were concealed, and the resulting network visualization was accomplished using Cytoscape3.7.2, a versatile platform for network analysis and visualization. For further analysis of core targets within the network, the CytoNCA plug-in was utilized. Nodes with degree and betweenness values exceeding the median in the PPI network were selected as potential core targets, which are deemed critical nodes in the network and serve as essential points for information transmission and efficiency.

These identified "key targets" hold paramount importance and will serve as the foundation for subsequent in-depth research, facilitating a comprehensive understanding of the molecular interactions and potential mechanisms underlying Danshenol C's pharmacological effects in PF. By employing this methodical approach, we aim to elucidate the key molecular players in Danshenol C's therapeutic actions, contributing valuable insights to the field of network pharmacology and guiding future investigations and therapeutic strategies for PF.

### GO analysis and KEGG pathway enrichment analysis

In order to comprehensively investigate the potential biological functions and signaling pathway mechanisms underlying the treatment of PF by Danshenol C, we performed GO enrichment analysis and KEGG pathway enrichment analysis on the common targets of Danshenol C and DEG. These analyses were conducted by employing the clusterProfiler, enrichplot, and org.Hs.eg.db software packages in R software. To ensure rigorous analysis and identify meaningful associations, we set the significance threshold at *P* < 0.05 for filtering, and further applied a corrected filtering condition of q = 0.05. By adhering to these stringent criteria, we aimed to focus on highly relevant pathways with statistically significant enrichment. Subsequently, relevant graphs were generated to visually represent the most pertinent pathways identified through the enrichment analysis. This graphical representation enabled a clear and concise portrayal of the molecular interactions and functions associated with Danshenol C's therapeutic effects in PF. To explore the biological function of the DEG targets, we analyzed the enrichment factor value. This allowed us to gain valuable insights into the functional implications of these genes and their potential roles in the pathogenesis of PF.

### Molecular docking verification

The structural characteristics of the core target protein were retrieved from the Uniprot database based on small resolution (Resolution) and the X-ray source (Method). Subsequently, the protein configurations were extracted from the RSCBPDB database to facilitate a comprehensive understanding of their spatial arrangements. To investigate the interactions between the core target and its active components at a molecular level, the 2D structures of these active components were acquired from the PubChem database. To ensure accurate and reliable predictions, the energy of these 2D structures was minimized using the Chem3D software.

Assessing the binding strength and activity of the active components with the core target was essential in characterizing their potential pharmacological effects. For this purpose, the SYBYL 2.0 software was employed to conduct rigorous evaluations. To visually elucidate the molecular interactions and key targets of the core active components among the entire set of active components, those with a total score (TotalScore) greater than 3 were carefully analyzed. The AutoDock 1.5.6 and PYMOL 2.4 software were instrumental in constructing visual representations of the molecular structures and key targets, providing critical insights into the molecular interactions and binding affinities.

By employing these sophisticated computational and visualization tools, we gained valuable insights into the structural aspects and binding characteristics of the core target and its associated active components. The integration of various software and databases in our methodology ensured accuracy and reliability in unraveling the molecular mechanisms underlying the interactions between the core target and active components. These findings are pivotal in advancing our understanding of the therapeutic potential of these active components and serve as a solid foundation for further investigation and development of targeted therapies in the realm of medicinal research.

## Experiment in vitro and in vivo of Danshenol C

### Reagents and equipment

Human peritoneal mesothelial cell line (HMrSV5) P4 was purchased from Guangzhou Genio Biotechnology Co., Ltd. Glucose-based peritoneal dialysis solutions (PDS) included 1.5%, 2.5% and 4.25% Dianeal, from Baxter Medical Co., Ltd. Danshenol C(HPLC purity > 98%, molecular formula: C21H20O4, molecular weight: 336.3811) was purchased from Nanjing Delge Biotechnology Co., LTD. E-cadherin, VAGF-A(BS6496), MAPK8(BS6448), MAPK14(BS3566), STAT3(BS1336), Caspase3(BS1518), P-P38(AF4001), P-STAT3(S727)(AP0248), P-STAT3 (T705)(AP0247) mouse anti-human monoclonal antibody antibodies were purchased from Santa Cruz. Instruments used: full-wavelength microplate meter (Thermo Varioskan LUX), constant temperature incubator (Thermo Scientific), frozen high-speed centrifuge (Eppendorf 5804R, 5418R), inverted light microscope (Leica DM1000 LED), automatic incubator (Bioworld, China), and Bio-Rad chemidoc XRS (Bio-Rad, USA).

### Cell viability

#### Effect of glucose concentration on HMrSV5 at different time periods

HMrSV5 cells in the logarithmic growth phase were cultivated for 24 h at 37℃in a 5%CO2 incubator. And then 100μL of clinical routine glucose peritoneal dialysate (containing 1.5%, 2.5%, 4.25% sugar, respectively) was added. 100μL basal medium was used as blank control group, with 4 rewells in each group. At selected time points (24 h, 36 h, and 48 h), CCK-8 solution was added to each well and incubated for 2 h. The absorbance at 450 nm was measured using a spectrophotometer.

#### Effects of different concentrations of Danshenol C on HMrSV5

From experiment 1, peritoneal dialysate containing 4.25% glucose was selected for subsequent experiments. HMrSV5 cells in the logarithmic growth phase were incubated for 24 h., Then Danshenol C was added with final concentrations of (0 μM, 10 μM, 20 μM, 40 μM, 80 μM, 160 μM). The DMEM complete medium containing 20% (v/v) fetal bovine serum and 1%100 U/mL penicillin/streptomycin was supplemented, and there were 4 re-wells in each group. After 48 h of co-culture, cck-8 solution was added for 2 h. The absorbance at 450 nm was measured using a spectrophotometer. According to Experiment 2, two groups of Danshenol C concentrations were selected for subsequent experiments. The final concentration of Danshenol in C1 group was 10 μM, and that in C2 group was 20 μM.

### Characteristics of HMrSV5 cell fibrosis

#### Morphological change characteristics

HMrSV5 cells in the logarithmic growth phase were seeded in a 6-well plate, and cultured with peritoneal dialysis containing 4.25% glucose for 48 h as the model group, and normal cells without peritoneal dialysis solution were set as the control group. In the drug treatment group, 25 μM Danshenol C was co-cultured with peritoneal dialysis containing 4.25% glucose for 48 h, and then observed at 20 magnification.

#### Expression of fibrosis-related proteins

HMrSV5 cells in logarithmic growth phase were seeded to confluent 80%. The old medium was removed and treated as follows. ①Normal group: 1 ml 20%DMEM complete medium + 1 ml DMEM basal culture; ②Control group: 1 ml 20%DMEM complete medium + 1 ml 4.25%peritoneal dialysate; ③Danshenol C 1:1 ml 20%DMEM complete medium + 1 ml 4.25%peritoneal dialysate (containing 10 μM Danshenol C); ④Danshenol C 2 group: 1 ml 20%DMEM complete medium + 1 ml 4.25%peritoneal dialysate (containing 20 μM Danshenol C). After 48 h of culture, western blot assay was performed. Three technical replicates were conducted for each sample. The dilution of Vascular endothelial growth factor-A(VEGF-A) and epithelial marker E cadherin (E-cadherin) was 1:1000, and the dilution of the corresponding secondary antibody was 1:5000.

### Total RNA extraction, cDNA preparation and qRT-PCR determination

Total RNA was extracted from each group by Trizol method, and cDNA was obtained by reverse transcription. The real-time PCR primers for MAPK8, MAPK14, CASP3, STAT3 and GAPDH were synthesized by Bgi Genomics Co., Ltd (Table 1). The reaction system contained 2 × SYBR Green PCR Master Mix 10μL, template cDNA(1:10 dilution) 5μL, each of upstream and downstream primers (10 um) 0.5μL, and deionized water 4μL. The amplification conditions were set as follows: predenaturation at 95℃ for 5 min, followed by 40 cycles of denaturation at 95℃ for 15 s and annealing at 60℃ for 30 s. The human GAPDH gene was used as an internal reference. ΔΔCt = (target gene-reference) Ct value—(control target gene-reference) Ct value; Relative mRNA expression = 2-ΔΔCt × 100%.

**Table 1:** Primer sequences

Genes	Forward primer	Reverse primer	RefSeq ID	Tm(F/R)	Amplicon size
JNK1 (MAPK8)	GACGCCTTATGTAGTGACTCGC	TCCTGGAAAGAGGATTTTGTGGC	NM_139049.4	61.11/61.07	136
p38 (MAPK14)	GAGCGTTACCAGAACCTGTCTC	AGTAACCGCAGTTCTCTGTAGGT	NM_001315.1	60.67/61.07	161
Caspase 3 (CASP3)	GGAAGCGAATCAATGGACTCTGG	GCATCGACATCTGTACCAGACC	NM_004346	61.54/60.80	146
STAT3	CTTTGAGACCGAGGTGTATCACC	GGTCAGCATGTTGTACCACAGG	NM_139276	60.68/61.45	133
GAPDH	AGAAGGCTGGGGCTCATTTG	GCAGGAGGCATTGCTGATGAT	NM_001256799	60.32/61.09	140
Fibronectin (FN)	ACAACACCGAGGTGACTGAGAC	GGACACAACGATGCTTCCTGAG	NM_212482	62.17/61.50	143
E-cadherin	GCCTCCTGAAAAGAGAGTGGAAG	TGGCAGTGTCTCTCCAAATCCG	NM_004360	60.87/62.57	131

### Total protein isolation and Western blotting

The cells were cultured and treated with drugs in the same way as above. After 48 h, proteins were extracted using whole protein extraction reagents (10ulPMSF, 10ul phosphatase inhibitor, and 1ul protease inhibitor in 1mlRIPA buffer). The supernatant was collected to measure the protein concentration by the BCA Protein Assay Kit. Add 1 × loading buffer to supernatant and heat with boiling water for 5 min. Equal amounts of protein were separated by electrophoresis on a 10% SDS-PAGE gel and then transferred to a PVDF membrane. Incubated with 5% skim milk in TBST for 1 h, and then mixed with primary antibodies against human E-cadherin, MAPK8(JUK1), MAPK14(P38), Stat3 (Y705), STAT3 (S727) and P-p38, Caspase3 and GAPDH were incubated overnight at 4℃. The membranes were then incubated with horseradish peroxidase labeled secondary antibody (IgG) for 1 h at 18℃. Band density was visualized using ECL Chemiluminescence detection and Bio-Rad ChemidOC XRS. Protein band densities were converted to gray values, and relative expression was expressed as gray values of target proteins normalized to GAPDH. Since little spacing above or below the target bands, the blots were cut prior to hybridisation with antibodies during blotting.

### Apoptosis

Cells were treated by group, 1 × 10^5^ cells were collected and resuspended in 200 μL Binding Buffer, adding 4 μL 0.5 mg/mL PI and 2 μL Annexin V-FITC solution; incubated at room temperature for 15 min; fluorescence was detected by flow cytometer.(488-nm laser excitation).

### Massone staining

After 1 week of adaptive rearing with about 30 g of C57BL/6 J mice, the normal group was given an intraperitoneal injection of an equal volume of normal saline, the control group received 3 ml of 4.25% peritoneal dialysate, experiment group received 4.25% peritoneal dialysate 3 ml (containing 20 μM Danshenol C). After 28 days of the above intervention, mouse peritoneal tissues were harvested for paraffin-embedded preservation to facilitate subsequent experiments.

After paraffin sections were removed, they were stained with hematoxylin, ponceau s and aniline blue, dehydrated by ethanol gradient, xylene transparent, and neutral gum seal and observed under the microscope.

### Immunohistochemical analyse

Paraffin-based sections were routinely processed. The antigen was repaired with Hydrogen Peroxide Block, blocked with 5% goat serum for 10 min after cooling, then E-cadherin and VEGF-A antibody were added and incubated for 4℃ overnight. Appropriate amount of biotin-labeled secondary antibody working solution was added by drip. After 37℃ light avoidance incubation for 30 min, color was developed with DAB chromogen for 3 min, then fully rinsed, counterstained, dehydrated, transparent, and sealed by tap water.

### Statistical analysis

Data were obtained from at least three independent experiments, described as mean ± standard error (SEM), and analyzed and plotted by GraphPad Prism9.0, Exor4 for XDMS_R, Image J, Photoshop, and other software. Differences between treatment groups were analyzed by t-test or ANOVA. Two-tailed *P* values < 0.05 were considered to indicate statistical significance.

## Results

### Screening of active ingredients, ADME analysis, and potential Target Prediction

**Fig. 2:**
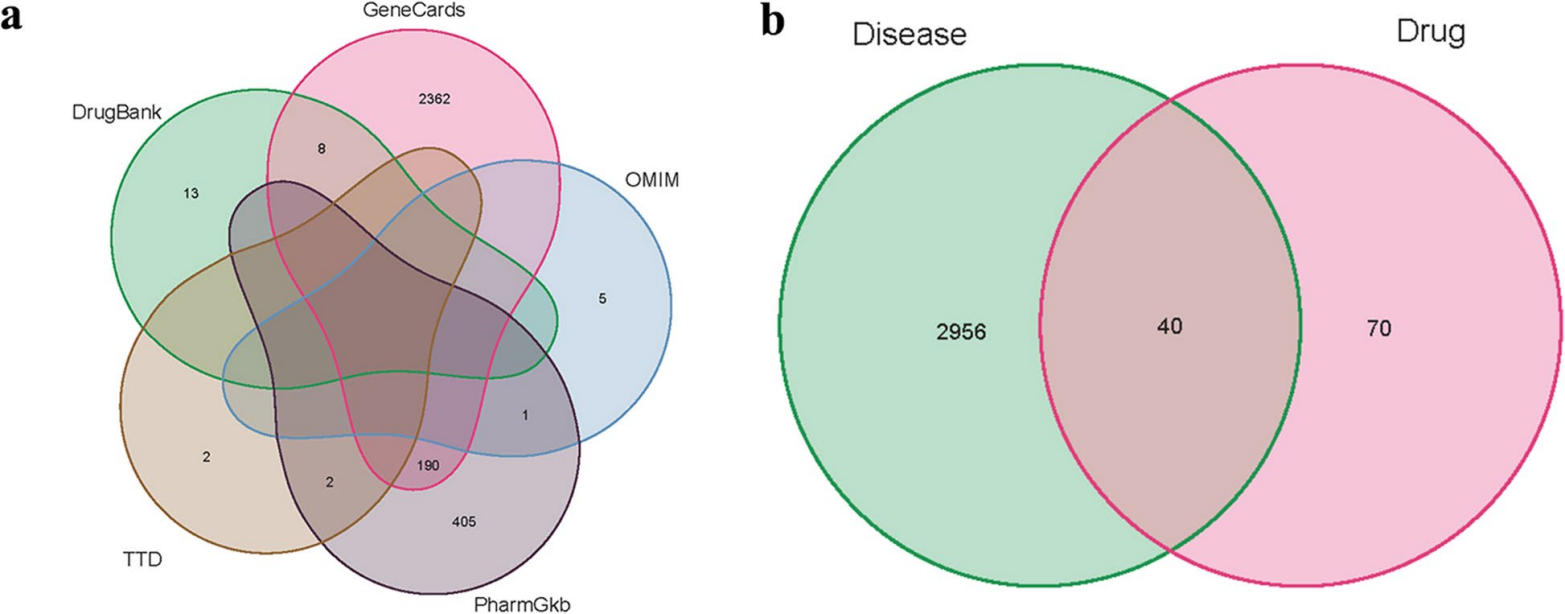
Venn diagram. **a** Five Disease datasets. **b** The targets in Disease and Danshenol C

Given the limited availability of target information pertaining to Danshenol C, it becomes imperative to prognosticate its potential targets based on its structural formula. The structural formula of Danshenol C was retrieved through meticulous screening using the YaTCM database. Subsequently, utilizing the Swiss Target Prediction database, a comprehensive repertoire of 43 active component species associated with Danshenol C was ascertained. A total of 110 drug targets were successfully identified. To ensure accuracy and consistency, the collated active ingredients and drug targets underwent meticulous refinement through Perl software and the UniprotKB database, thereby securing their official gene names. In pursuit of a holistic understanding, the amalgamation of genes present in the aforementioned databases was undertaken (Fig. 2a). To attain a sufficiently robust dataset catering to PF, differential genes sourced from GEO and genes procured from the aforementioned databases were harmoniously integrated. The subsequent step involved a meticulous alignment of disease genes with corresponding drugs (Table 2 and Fig. 2b).

**Table 2:**
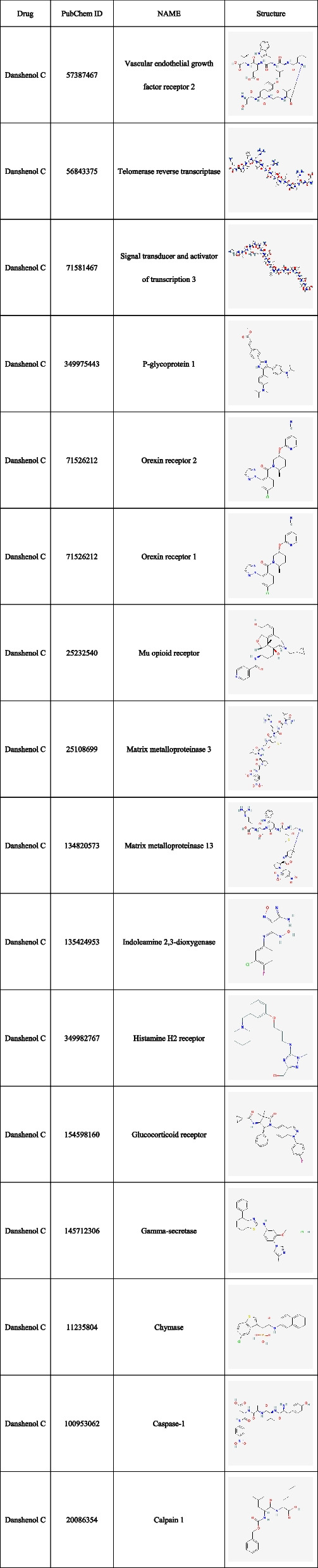
Core components of Danshenol C

### Screening, batch-normalization correction, and analysis of disease-associated targets in PF

**Fig. 3:**
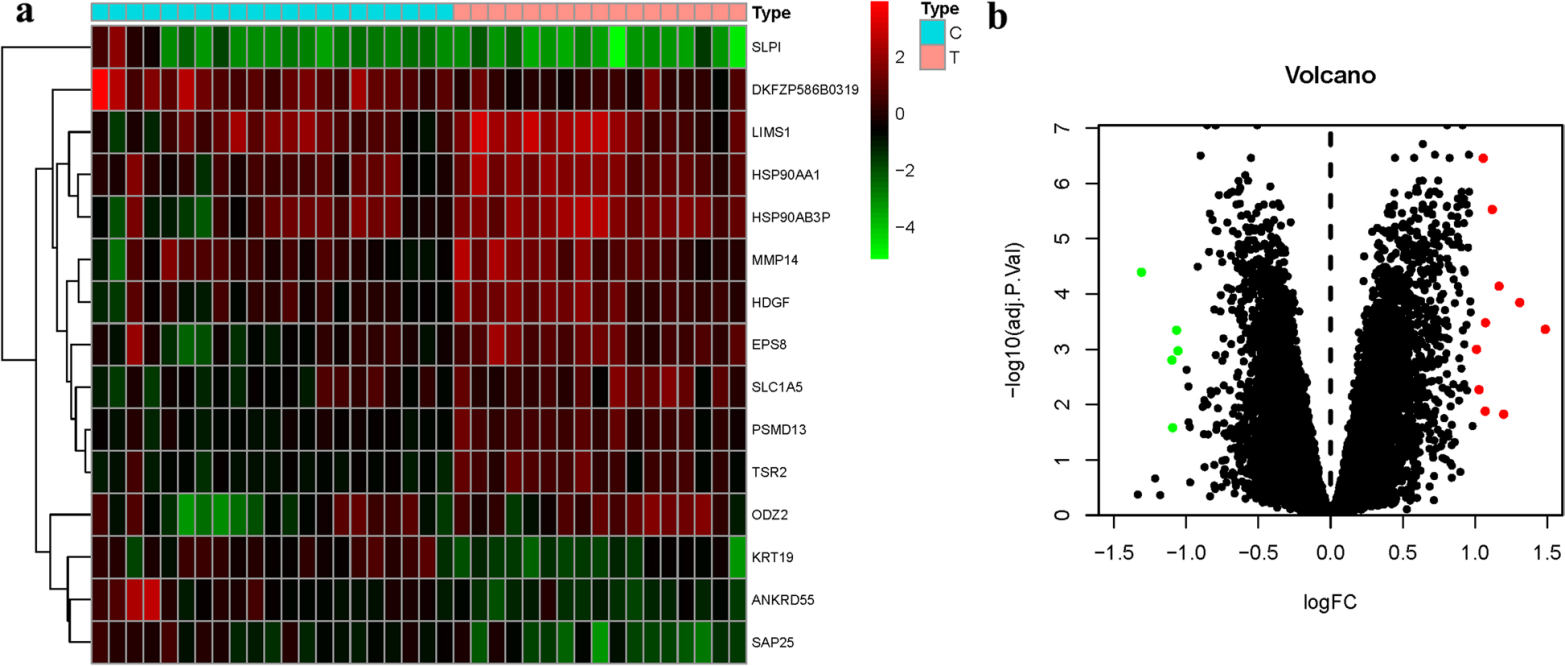
Differential genes volcano and heat map of GEO. **a** Heatmap. **b** Volcano map

**Fig. 4:**
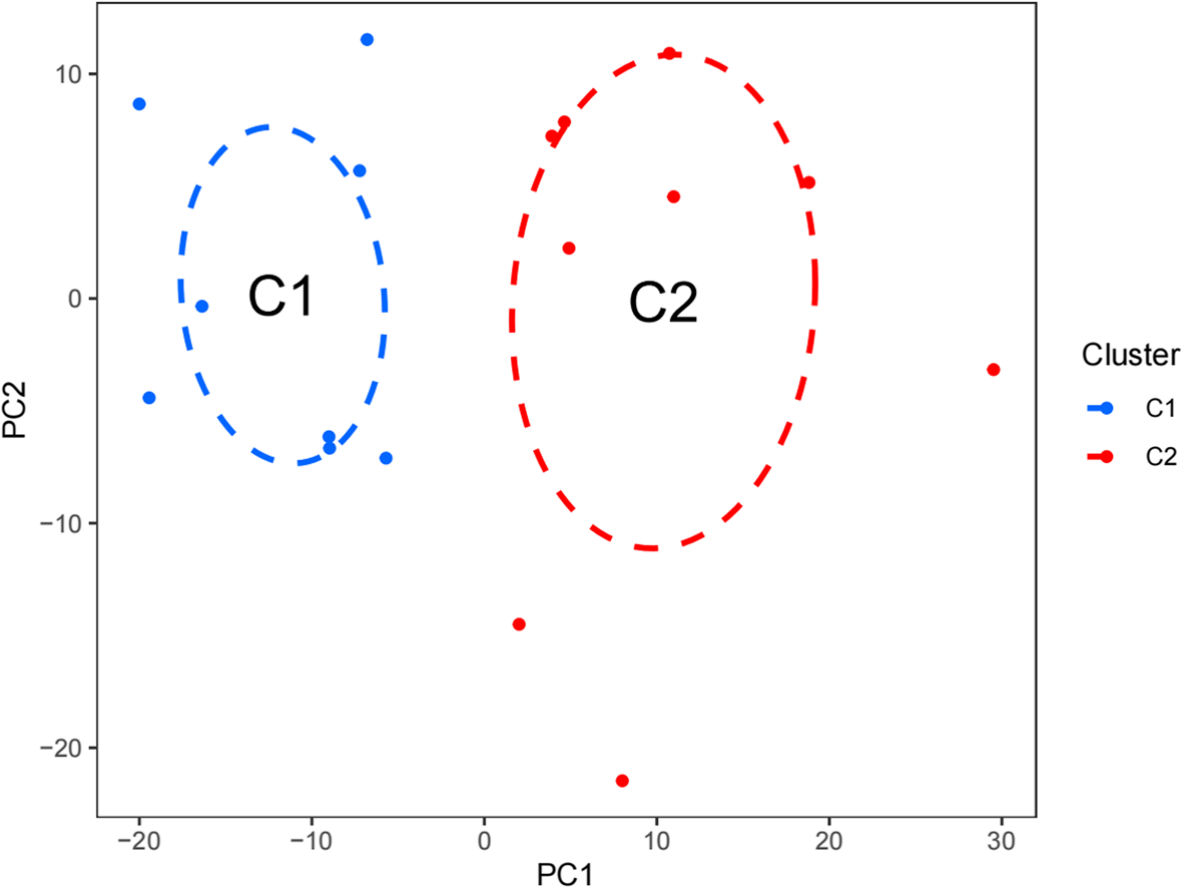
A PCA plot

**Fig. 5:**
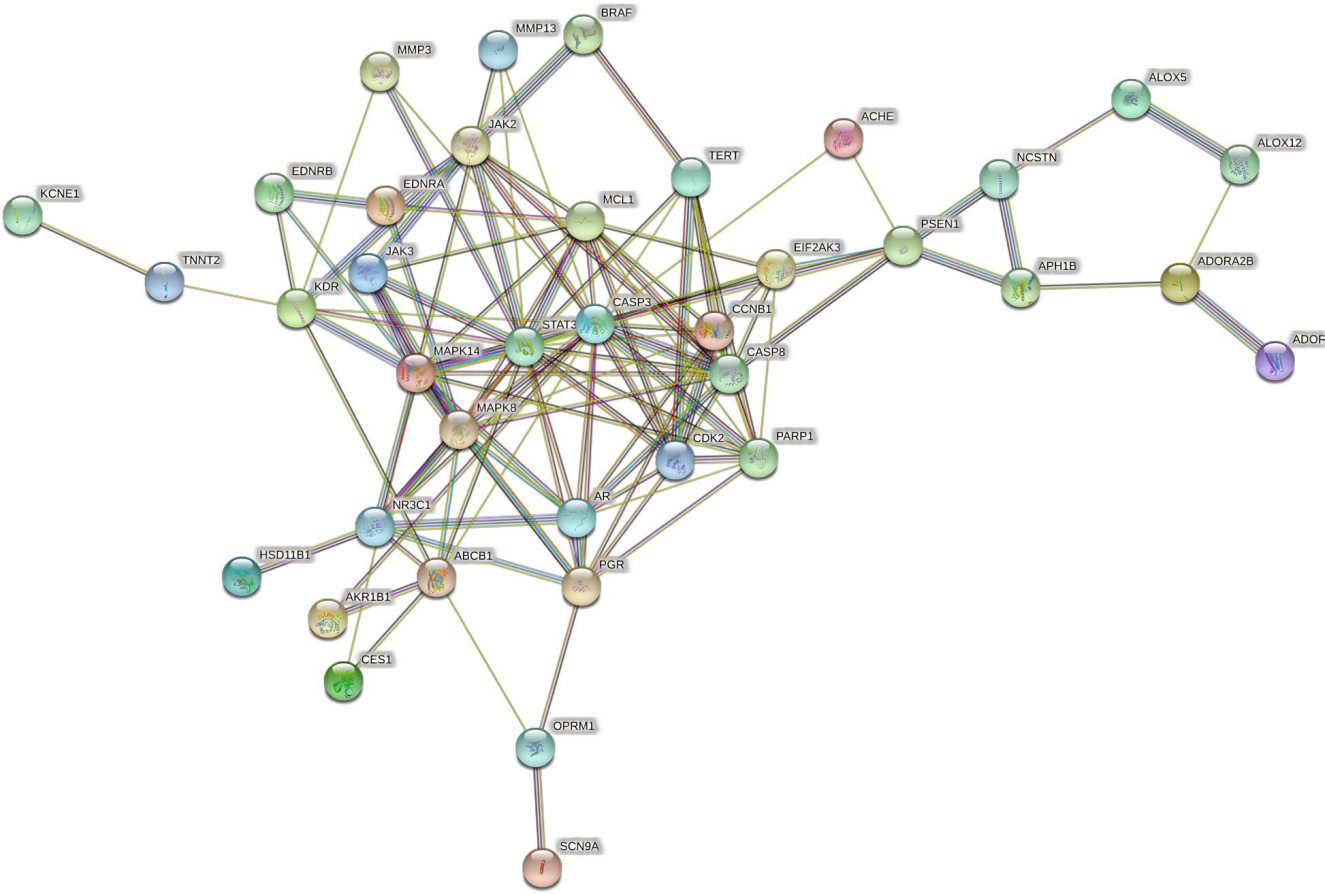
Herb-ingredients-targets (H-I-T) network

The GSE92453 dataset, encompassing 17 samples of Peritoneal Membrane and 21 samples of Omentum, was meticulously procured from the GEO chip database. To ensure data integrity and comparability, the "SVA" package, a powerful tool within the R language, was adeptly deployed to execute batch normalization on the two acquired datasets. Following the correction process, an assemblage of 15 DEGs was successfully identified from the two distinct groups, skillfully facilitated by the "Limma" package within the R language. Among these identified DEGs, 10 genes (66.66%) displayed up-regulation, while 5 genes (33.33%) exhibited down-regulation. A graphical representation of the differentially expressed genes is visually depicted in Fig. 3. Based on the PCA findings, patients with differing risks were separated into two groups (Fig. 4). Furthermore, an insightful depiction of the intricate interplay between 40 drugs and the genes associated with the disease phenotype was meticulously illustrated in Fig. 5.

### Construction of PPI network and identification of key targets

**Fig. 6:**
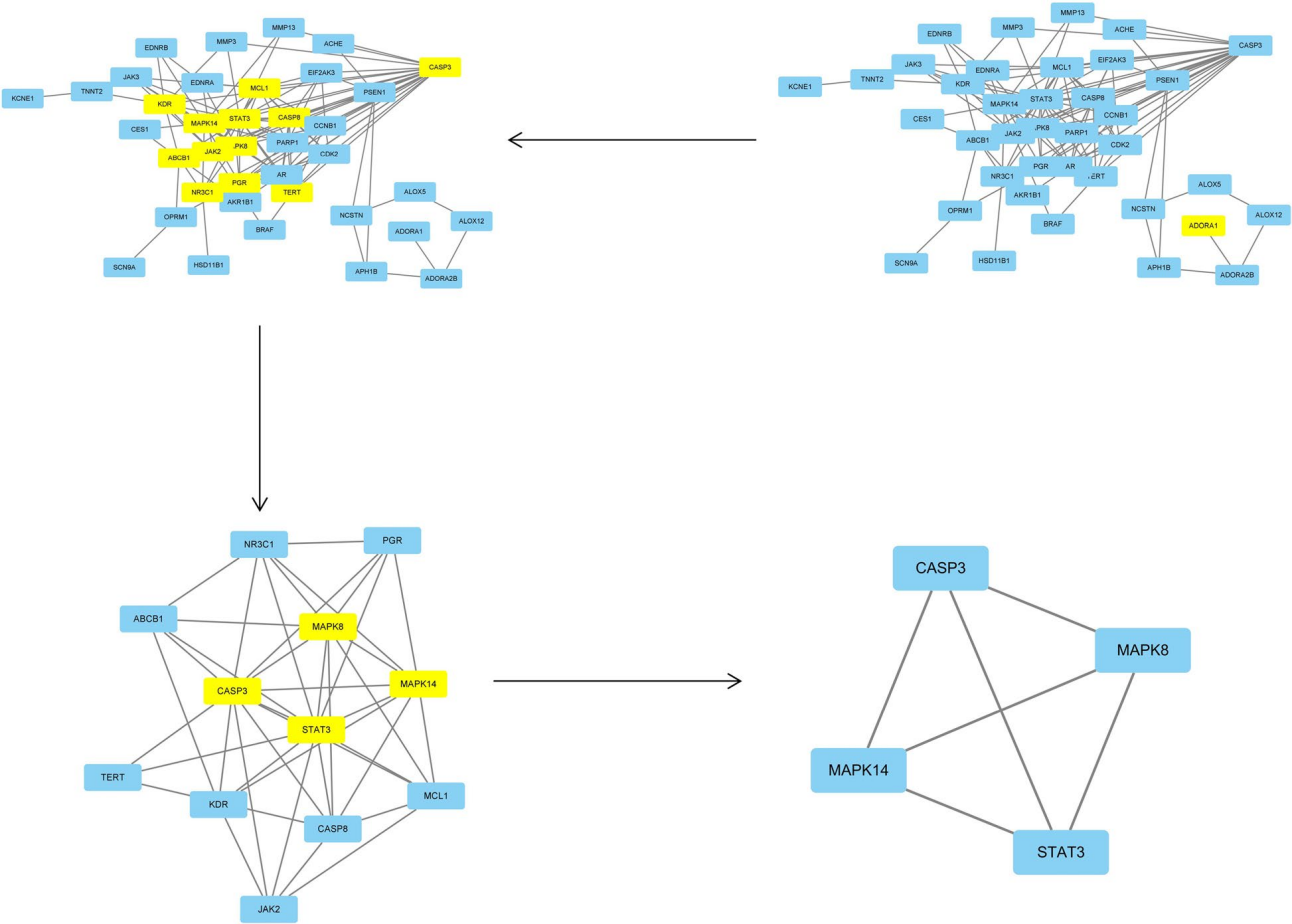
Target screening strategy diagram of Danshenol C in the treatment of PF key nodes

Cytoscape3.7.2 software was used to obtain a PPI network map of 40 related targets and their relationships (Fig. 5). Then, the intersection target interaction relationship calculated from String database was visualized, and the intersection of the above PPI network map was extracted with CytoNCA toolkit. After screening twice, STAT3, MAPK14, MAPK8, CASP3 were found to be the main efficacious genes (Fig. 6).

### GO and KEGG enrichment analysis

**Fig. 7:**
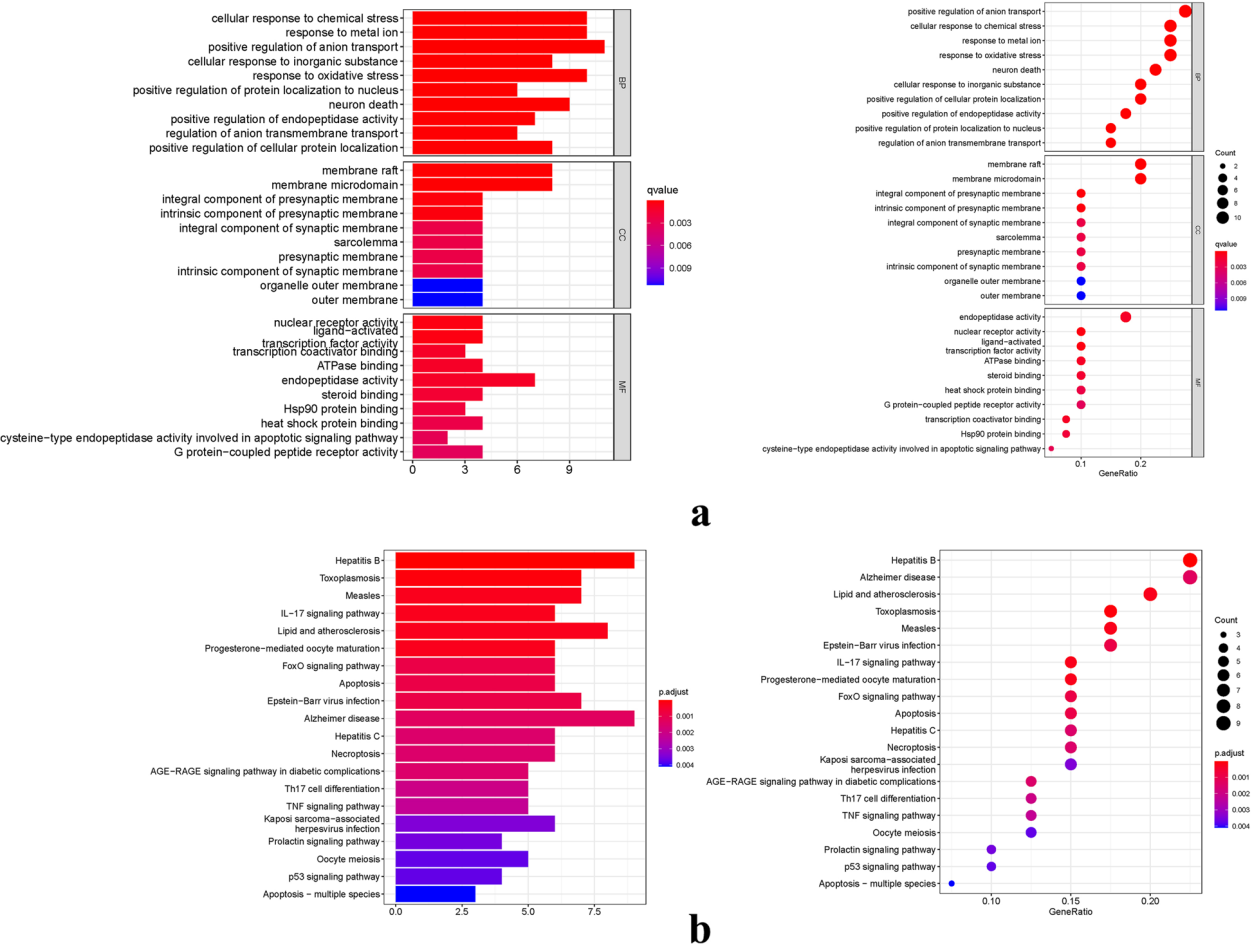
GO and KEGG analysis of potential targets of Danshenol C in the treatment of PF (**a**): The GO barplot and bubble illustrates the scatter map of the selected gene's logFC. **b**: The KEGG barplot and bubble illustrates the scatter map of the logFC of the indicated gene

**Fig. 8:**
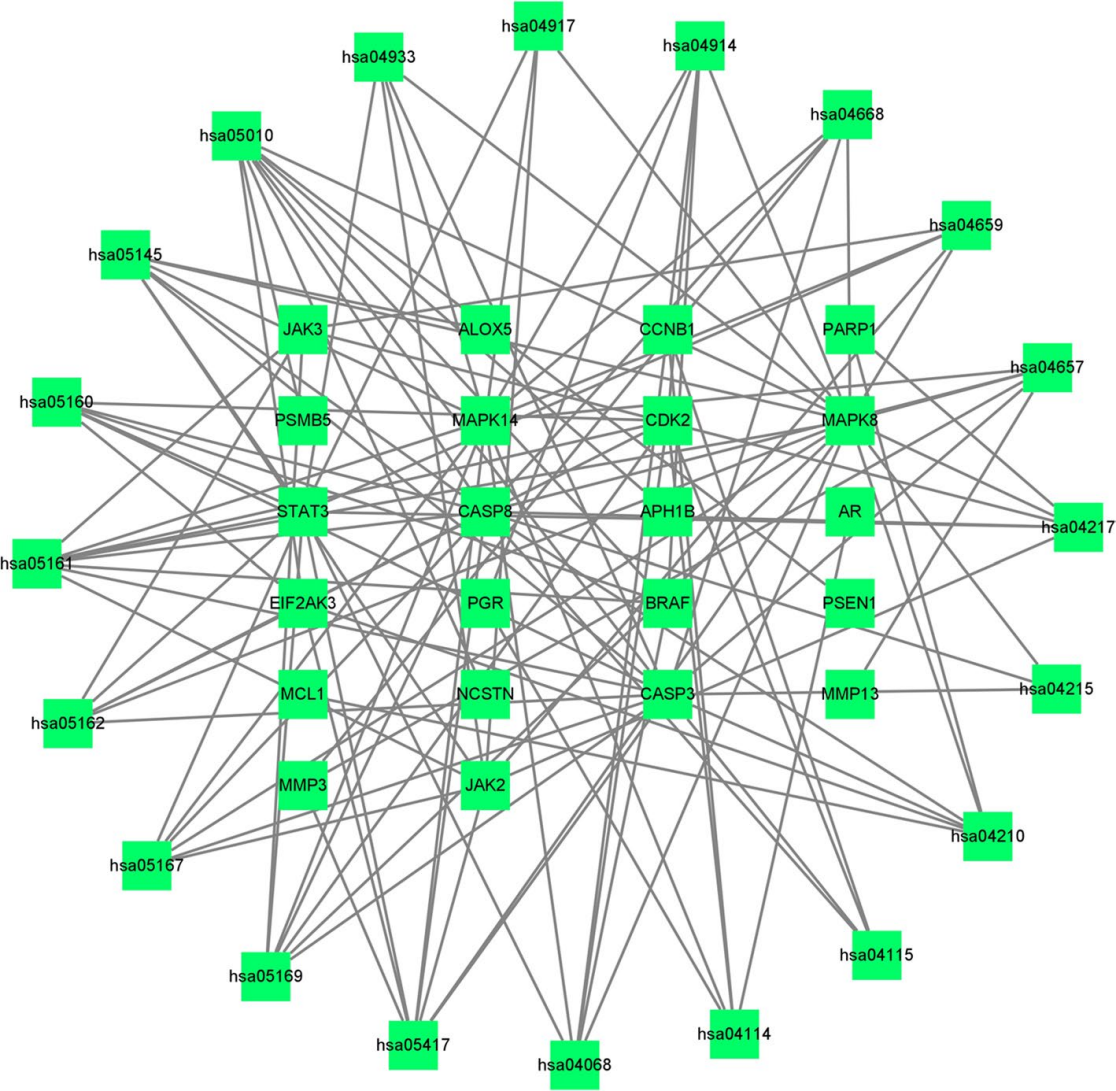
KEGG analysis of the target-pathway network. The edges reflect the interactions between the targets and the paths, and the node size is proportional to the degree of connection

The GO analysis performed in this study led to the identification of 948 core targets, encompassing MF, CC, and BP. The MF category predominantly involved passive transmembrane transporter activity (GO:0022803), channel activity (GO:0015267), and DNA-binding transcription activator activity (GO:0001216). Within the CC category, significant associations were found with synaptic membrane (GO:0097060), chromosomal region (GO:0098687), and membrane raft (GO:0045121). As for BP, key involvement was observed in neutrophil activation (GO:0042119), divalent inorganic cation homeostasis (GO:0072507), and neutrophil-mediated immunity (GO:0002446). Moreover, the utilization of KEGG analysis allowed us to discern the primary signaling pathways. The overexpressed genes were found to be predominantly associated with the Pathways of neurodegeneration-multiple diseases (hsa05022), Alzheimer's disease (hsa05010), and Neuroactive ligand-receptor interaction (hsa04080) (Fig. 7). To enhance clarity and comprehension, the data results of KEGG were effectively visualized (Fig. 8).

### Molecular docking

**Fig. 9:**
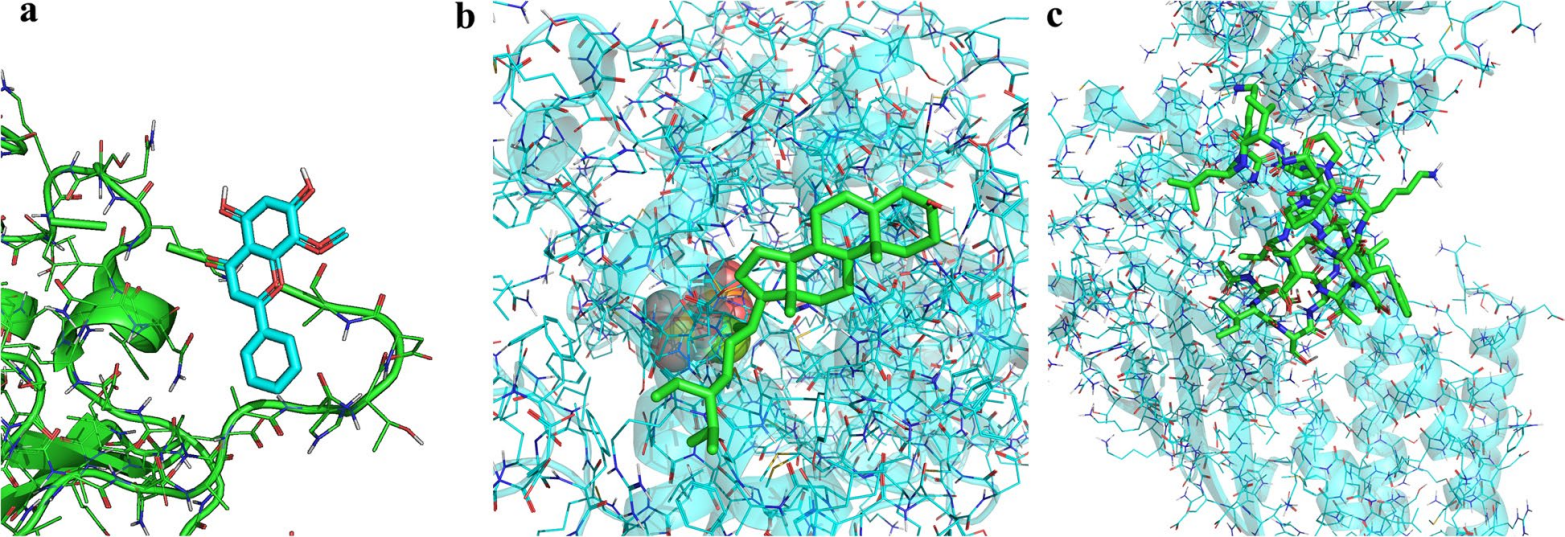
Molecular docking diagram of molecular structures and key targets (**a**) MAPK14-Wogonin. **b** MAPK8-sitosterol. **c** STAT3-STAT5

To unravel the molecular interactions and binding affinities of the core targets extracted from the esteemed Uniprot database (namely, MAPK14 with Wogonin, MAPK8 with sitosterol, and STAT3 with STAT5), we employed the advanced SYBYL2.0 software. By doing so, we were able to thoroughly assess the binding strength and activity of these protein–ligand complexes (Table 3). In the pursuit of a deeper understanding of the intricate molecular associations, we further conducted molecular docking using the eminent AutoDock1.5.6 and PYMOL2.4 software. This elaborate process allowed us to precisely position the molecular structures of the key active components in relation to the core targets. The outcomes of these docking simulations, as showcased in Fig. 8, elegantly elucidated that Wogonin, Sitosterol, and STAT5 emerged as the principal small molecule active components and gene targets of Danshenol C in the therapeutic intervention against PF (Fig. 9).

**Table 3:** Binding energy of key active components of Danshenol C in the treatment of PF and key target docking

Component	Chemical formula	Relative molecules	Target	Binding energy/(kj·mol^−1^)
Wogonin	C_16_H_12_O_5_	284.26 g/mol	MAPK14	-9.7
sitosterol	C_15_H_10_O_6_	286.24 g/mol	MAPK8	-9.4
Signal transducer and activator of transcription 5	C_109_H_182_N_26_O_32_	2368.8 g/mol	STAT3	-7.8

### High glucose peritoneal dialysate inhibits HMrSV5 activity

**Fig. 10:**
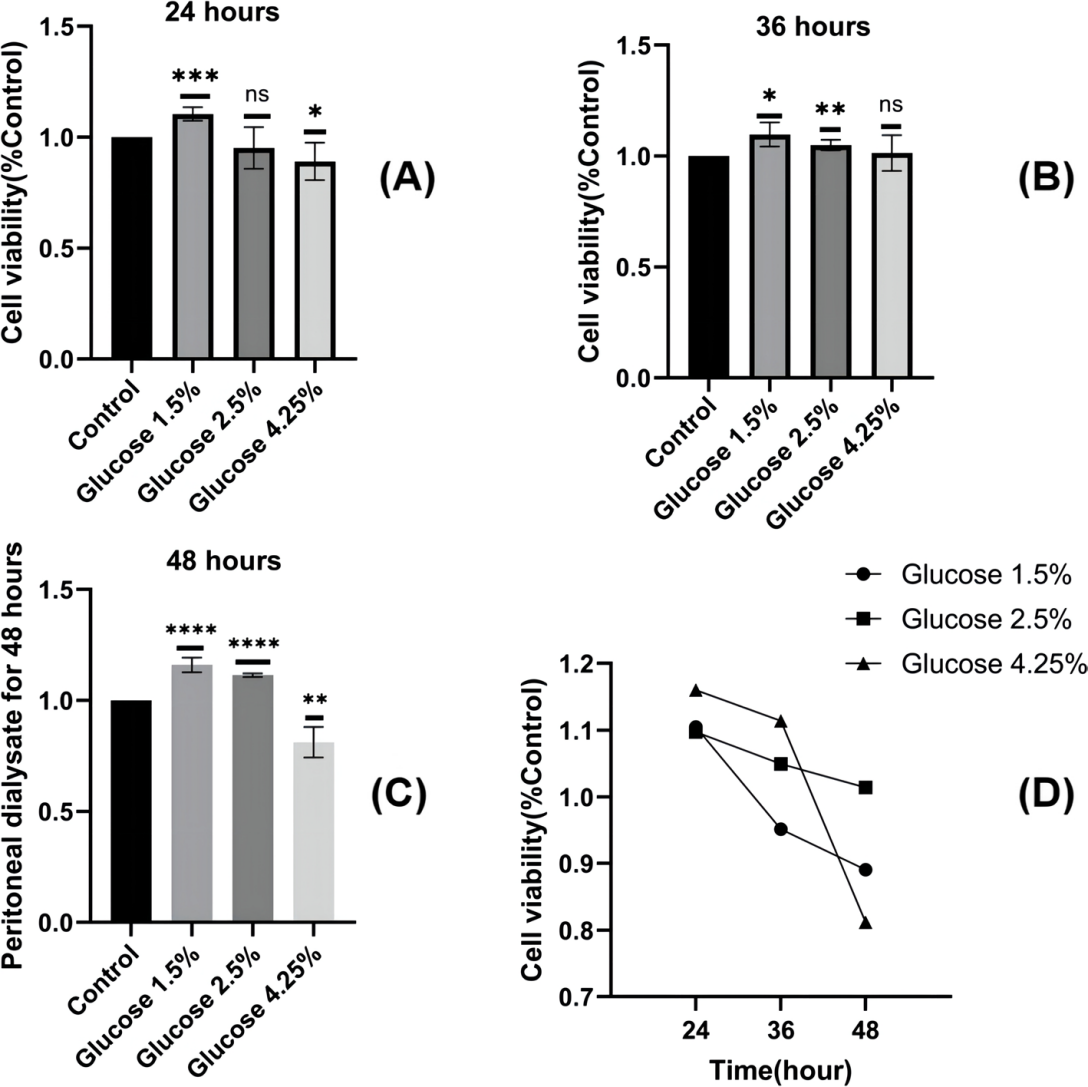
Effect of PDS on HMrSV5 activity **A** Different concentrations of PDS (1.5% Dianeal, 2.5% Dianeal, 4.25% Dianeal) were used for 24 h; **B** Treatment with 4 concentrations of PDS for 36 h; **C** Treatment with 4 concentrations of PDS for 48 h; **D** Comparison chart at different times; ns: *P* > 0.05; *: *P* < 0.05; **: *P* < 0.01; ***: *P* < 0.001; ****:*P* < 0.0001

In order to select the appropriate concentration and time for membrane construction, we selected three specifications of clinically commonly used sugar-containing peritoneal dialysate (1.5%, 2.5%, 4.25%) and performed cytotoxicity assay at different time points (24 h, 36 h, and 48 h). According to CCK-8 assay, compared with the normal group, only 4.25%PDS inhibited the cell viability at all three time points, and significantly decreased the cell viability at 48 h. (Fig. 10A, B, C, D) The results showed that 4.25%PDS could significantly inhibit the activity of HMrSV5 for 48 h, and 4.25%PDS was selected for subsequent experiments.

### Danshenol C reversed the inhibition of HMrSV5 activity under high glucose condition

**Fig. 11:**
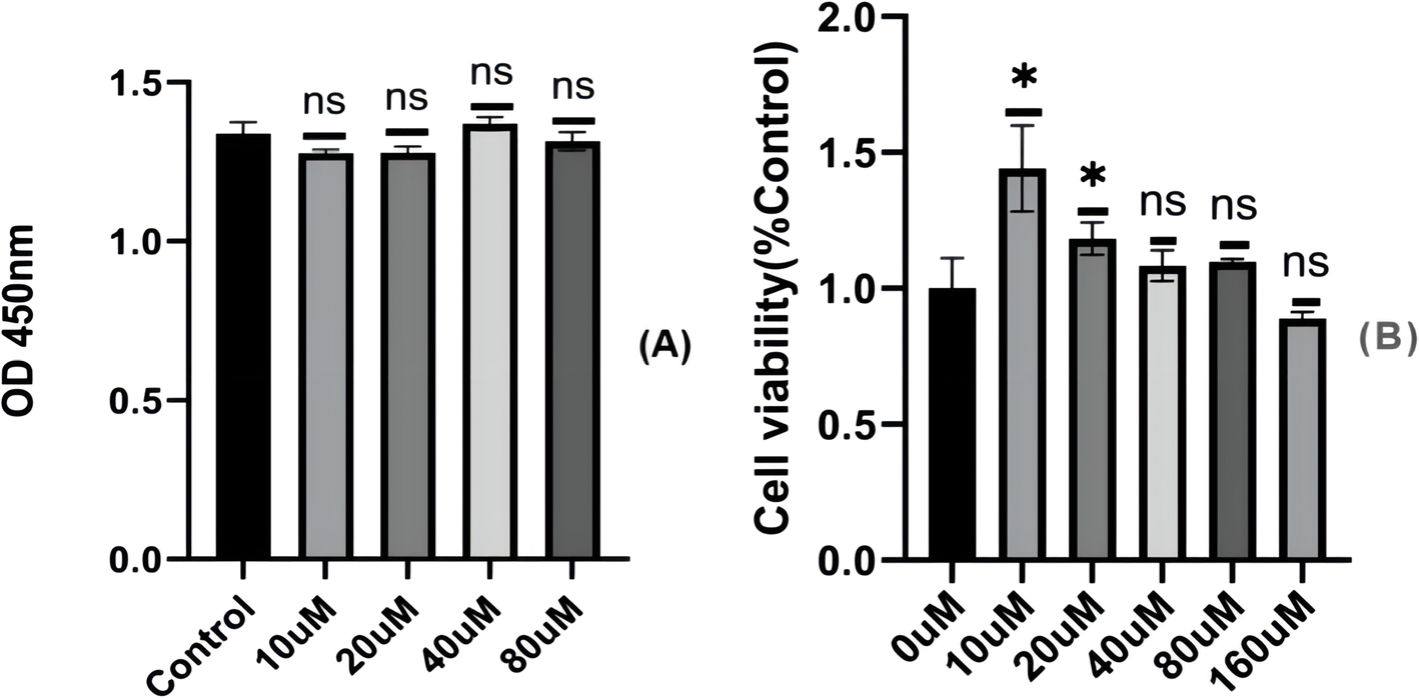
The toxicity of Danshenol C to HMrSV5 cells. **A** Treatment with different concentrations of Danshenol C for 48 h; **B** Different concentrations of Danshenol C + 4.25%PDS for 48 h ns: *P* > 0.05; *: *P* < 0.05

In order to select the appropriate intervention dose of Danshenol C, we carried out cytotoxicity assay in HMrSV5 cells. We first treated cells with different concentrations of Danshenol C (5, 10, 15, 20, 25, 40 and 80 μM) alone, and found that the viability of HMrSV5 cells did not change after 48 h of intervention (Fig. 11 A). Then we cocultured with different concentrations of Danshenol C (10,20, 40, 80 and 160 μM) and 4.25% high glucose peritoneal dialysate for 48 h. According to CCK-8 assay, 10, 20, 40 and 80 μM Danshenol C promoted the cell viability after high glucose treatment, while 160 μM Danshenol C slightly decreased the cell viability (Fig. 11B). The results showed that 10 and 20 μM Danshenol C could significantly reverse the PDS-induced decrease of HMrSV5 cell viability (*P* < 0.05). To verify the subsequent experiments, we selected two groups of Danshenol C concentrations. The final concentration of Danshenol group C1 was 10 μM, and the final concentration of Danshenol group C2 was 20 μM.

### Danshenol C can improve fibrosis on morphology and fibrosis markers

**Fig. 12:**
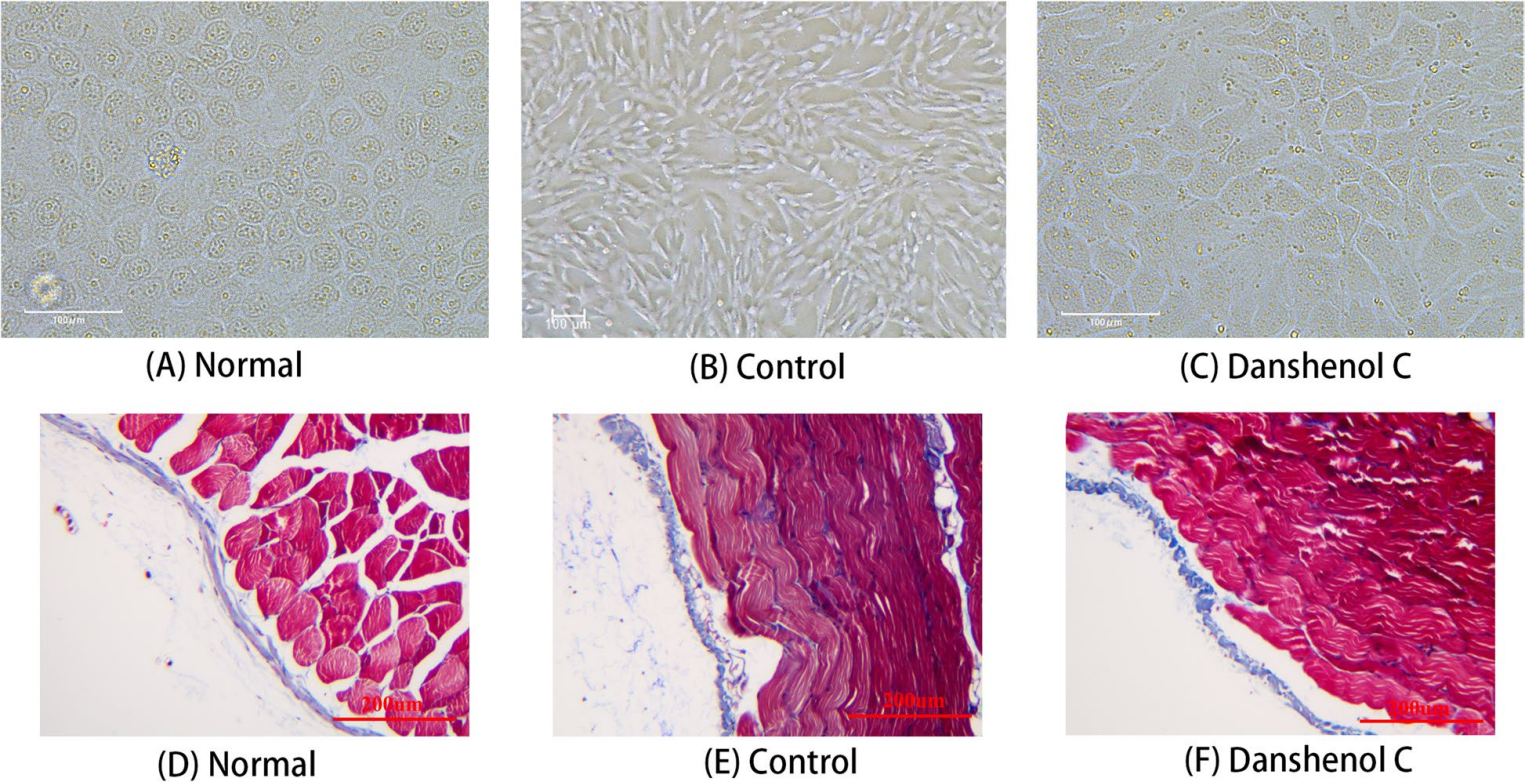
Effects of Danshenol C on morphology of cell and tissue **A** normal cells; **B** cells treated with 4.25%PDS for 48 h; **C** Danshenol C + 4.25%PDS for 48 h (**D**) normal peritoneal tissue of mice; **E** intraperitoneal injection 4.25%PDS for 28 days; **F** intraperitoneal injection Danshenol C + 4.25%PDS for 28 days

According to the microscopic observation, the normal cells were round and oval, and the cells were spindle shaped after 48 h of 4.25%PDS treatment, and the cell image was in the middle of the two after drug addition (Fig. 12A, B, C).

Furthermore, normal peritoneal tissue was smooth and mesothelial dense according to Massone staining. Mice treated with peritoneal dialysis fluid had deposited collagen fibers in the peritoneal interstitial layer with an enlarged submesothelial dense zone associated with increased inflammatory cells. The presentation of peritoneal histomorphology after Danshenol C intervention was intermediate between the two groups (Fig. 12D, E, F).

**Fig. 13:**
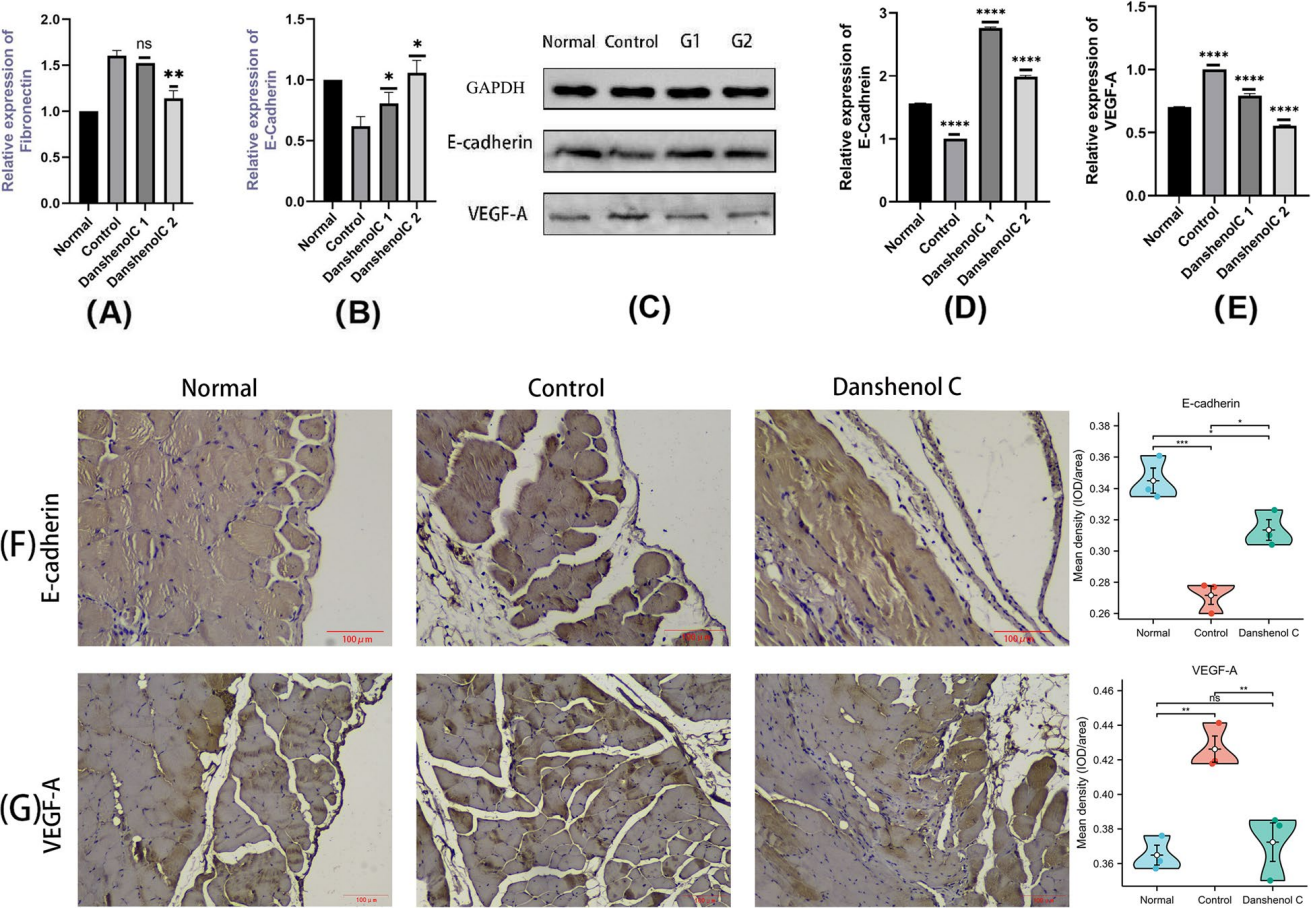
Effects of Danshenol C on fibrosis markers (**A**)(**B**) mRNA expression; **C** western blot; **D** (**E**) protein expression;(**F**)(**G**) histochemistry ns: *P* > 0.05; *: *P* < 0.05; **: *P* < 0.01; ***: *P* < 0.001; ****: *P* < 0.0001

Real-time RT-PCR showed that compared with the control group, the mRNA expression of Fibronectin in the Danshenol C1 and C2 groups was decreased (Fig. 13A), and the mRNA expression of E-cadherin was significant increased (Fig. 13B), and the increase was more significant in the high concentration of Danshenol C.

Western blot analysis showed that high glucose induced Epithelial-mesenchymal transition(EMT) in peritoneal mesothelial cells. Compared with normal cells, VEGF-A protein expression was increased and E-cadherin protein expression was decreased. The VEGF-A protein expression was decreased and E-cadherin protein expression was decreased in the two groups after Danshenol C treatment. As shown in Fig. 13C, D, E.

Immunohistochemical analysis of peritoneal tissue in mice showed that, after treated with 4.25% PF, VEGF-A positive area increased and E-cadherin-positive area decreased compared with normal peritoneal tissue. VEGF-A positive area decreased after Danshenol C intervention and increased in E-cadherin positive area. As shown in Fig. 13F, G.

### Potential pathways and mechanisms of Danshenol C reversal of hyperglycemic fibrosis

**Fig. 14:**
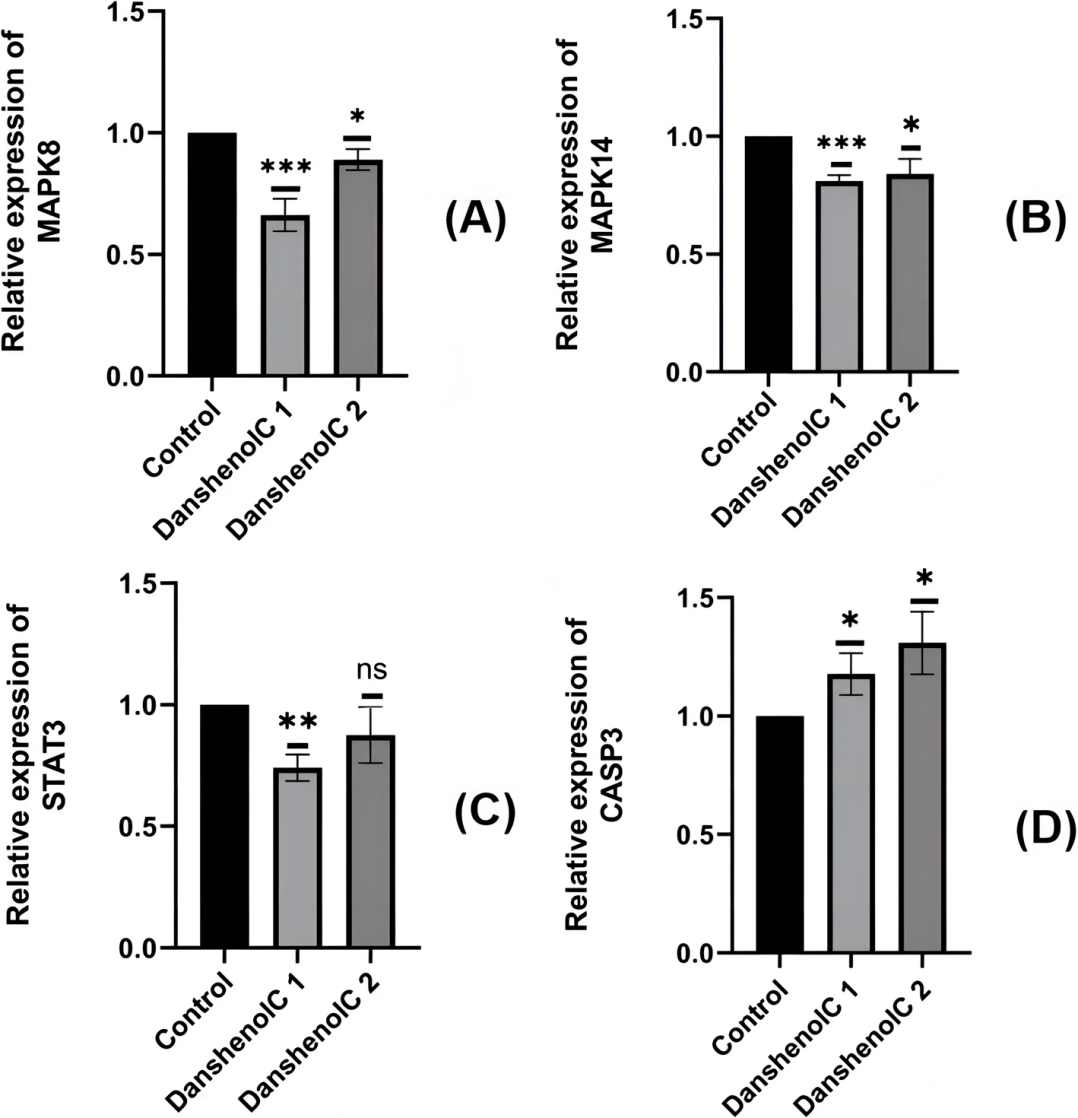
Relative RNA expression levels of the target genes (**A**) (**B**) Compared with the control group, the C1 C2 groups were significantly decreased; **C** Compared with the control group, group C1 was significantly decreased, while group C2 had no significant change; **D **Compared with the control group, the C1 C2 group increased significantly; *:*P* < 0.05; **:*P* < 0.01; ***: *P* < 0.001

Through the above network pharmacology, we found four target genes related to Danshenol C and PF, namely MAPK8 (JNK1), MAPK14 (P38), CASP3 and STAT3. Real-time RT-PCR showed that compared with the control group, the mRNA expression of MAPK8 in the C1 and C2 groups was significantly decreased (Fig. 14A), and the mRNA expression of MAPK14 was decreased (Fig. 14B), and the decrease was more significant in the low concentration of Danshenol C. Compared with the control group, the mRNA expression of STAT3 in the Danshenol C1 group was decreased, but there was no difference in the Danshenol C2 group (Fig. 14C). Compared with the control group, the mRNA expression of CASP3 of Danshenol C increased significantly. (Fig. 14D).

**Fig. 15:**
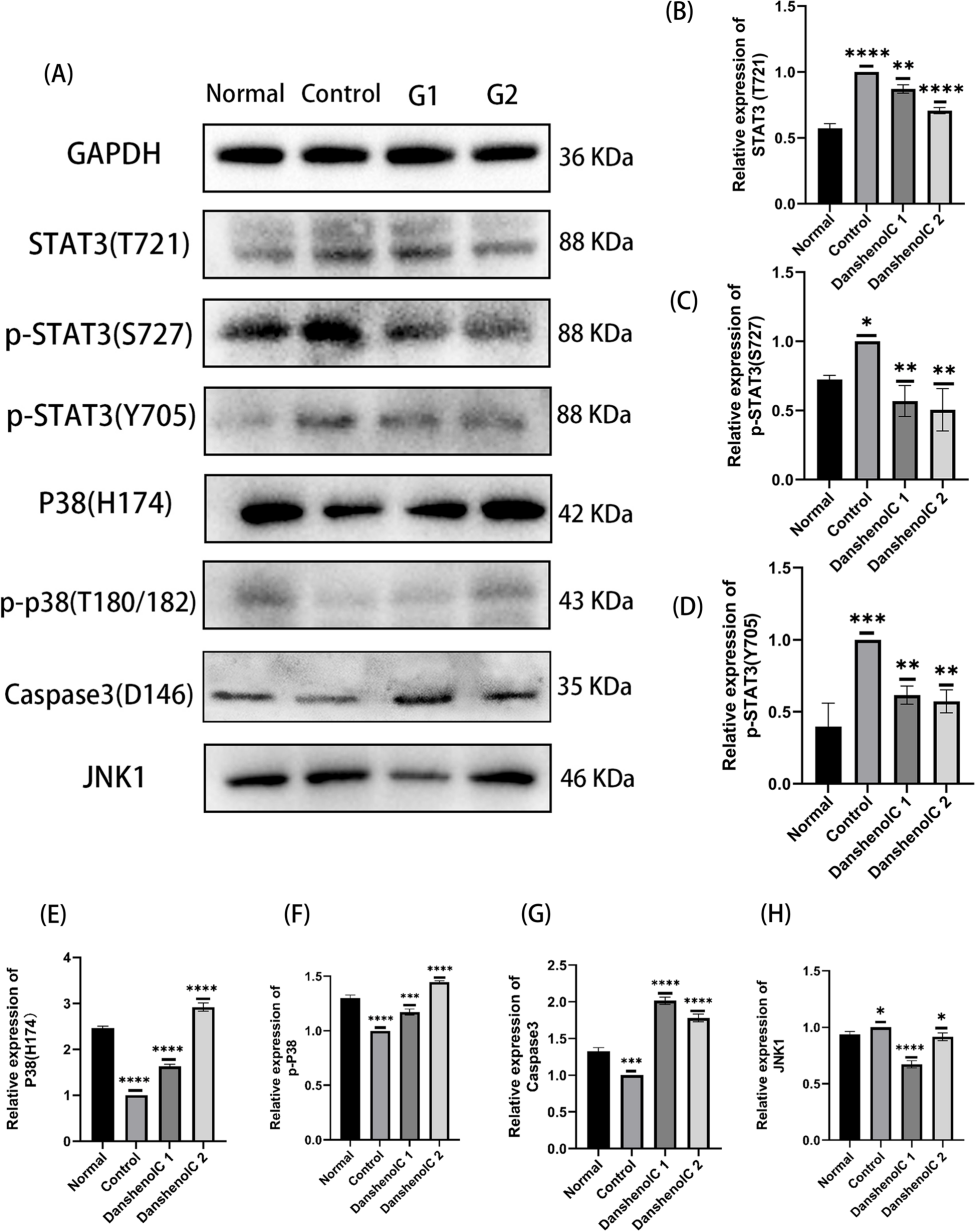
Relative protein expression levels of the target genes (**A**): Western blot images; **B** (**C**)(**D**)(**H**): Compared with the normal group, the expression level of target protein in the control group was significantly increased; Compared with the control group, the Danshenol C group decreased significantly; **E** (**F**)(**G**): Compared with the normal group, the expression level of the target protein in the control group was significantly decreased; Compared with the control group, the Danshenol C group increased significantly; *:*P* < 0.05; **:*P* < 0.01; ***: *P* < 0.001; ****: *P* < 0.0001

To further explore the genes involved and possible pathways, Western blotting was performed to measure the protein expression levels of STAT3(T721), STAT3(S727), STAT3(T705), MAPK14 (P38 (H174)), P-p38 (T180/182), Caspase3 and MAPK8 (JNK1). The blot of the protein band is shown in Fig. 15A. Compared with normal cells, the protein levels of P-P38, P38 and Caspase3 in fibrotic cells were decreased (Fig. 15E, F, G) (*P* < 0.05), indicating that the expression of these three genes was inhibited. Compared with normal cells, the protein expression level of STAT3 and JNK1 in fibrotic cells was increased (Fig. 15B, C, D, H) (*P* < 0.05). Compared with fibrotic cells, the protein levels of P-P38, P38 and Caspase3 in Danshenol C treated cells were significantly increased (Fig. 15E, F, G) (*P* < 0.05), and the protein expression level of STAT3 and JNK1 was significantly decreased (Fig. 15B, C, D, H) (*P* < 0.05).

**Fig. 16:**
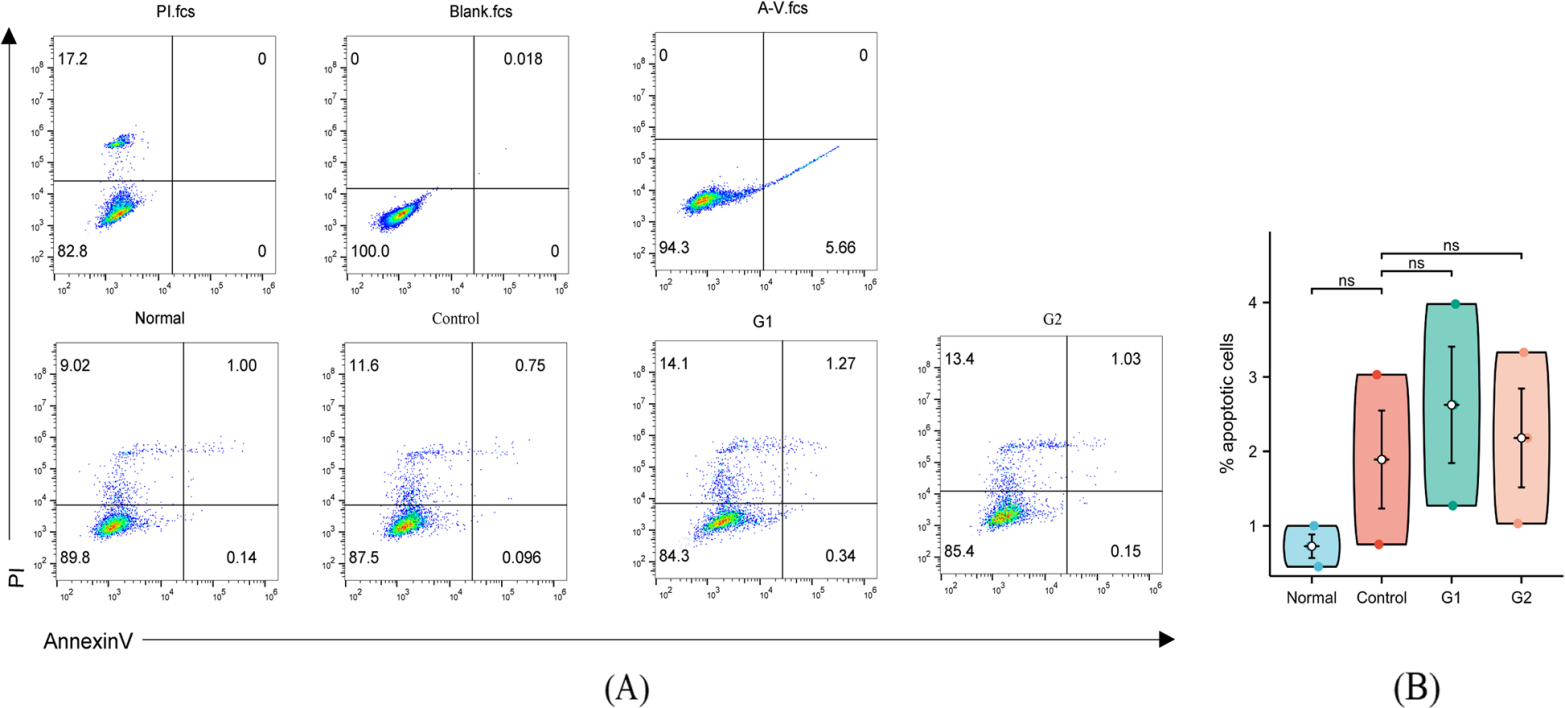
Cell apoptosis ns: *P* > 0.05

Caspase3 Is closely related to apoptosis. We conducted apoptosis flow analysis and found that apoptotic cells increased compared with control and model groups, but there was no statistical difference (Fig. 16AB) (*P* > 0.05).

## Discussion

At present, there is a lack of effective means to intervene PF. To deeply explore the mechanism of peritoneal dialysis-related PF and to find drugs to intervene PF has become an important measure to improve the diagnosis and treatment of peritoneal dialysis. Previous studies have found that *astragalus membranaceus, Panax notoginseng, salvia miltiorrhiza, ligusticum Chuanxiong, emodin* and other drugs can inhibit the occurrence and progression of PF and protect the morphology, structure and function of peritoneal tissue by inhibiting or antagonizing inflammatory factors and pro-fibrotic cytokines [15–19]. In Abdali's study, they have discovered that both ursolic acid and Kamolonol Acetate exhibit cytotoxic effects on the HCT116 cell line. Moreover, it has been observed that ursolic acid may potentially enhance the radiosensitivity of human colorectal tumor cells through the NF-κB1 and CCND1 signaling pathways [20]. Most of them are single medicine or Chinese patent medicine injection, involving multiple components, so it is difficult to elaborate the related mechanism. Therefore, this study investigated the therapeutic mechanism of Danshenol C on PF.

In our investigation, we observed that four proteins, namely STAT3, MAPK14, MAPK8, and CASP3, held the highest degrees of protein interaction. Extensive research has underscored the pivotal role of MAPK as a key signaling pathway governing a myriad of cellular processes. Crucially, MAPK serves as a vital mediator, transmitting signals from cell surface receptors to the nucleus's DNA. As a result, it orchestrates diverse biological phenomena, encompassing cell growth, apoptosis, and the regulation of the cell cycle. Additionally, MAPK plays an instrumental role in modulating pathological processes, including inflammation, autophagy, and stress response. Notably, MAPK14 emerges as an indispensable member within the MAPK family, signifying its significance in cellular regulation [21, 22]. STAT3 is a transcription factor that can be activated by MAPK and plays a key role in many cellular processes, such as cell growth and apoptosis, and is involved in the occurrence and development of tumors [23]. Furthermore, it is noteworthy that STAT3 plays a critical role in the differentiation of TH17 helper T cells and is intricately linked to autoimmune diseases, recurrent infections, and other pathological conditions [24, 25]. Evidence suggests that the upregulation of phosphorylated STAT3 in peritoneal mesothelial cells, as well as the activation of hypoxia-inducible factor-1α (HIF-1α) in peritoneal mesothelial cells, among patients undergoing long-term peritoneal dialysis, can trigger the process of epithelial-to-mesenchymal transition (EMT) or induce apoptosis of peritoneal mesothelial cells [26]. Caspase 3 (CASP3) and Caspase 8 (CASP8) are critical proteases involved in apoptosis. Regulation of CASP3 and CASP8 has been demonstrated to attenuate cell proliferation and migration [27–29]. Additionally, investigations have revealed that deficiencies in CASP3 may result in altered kidney and immune organ functionality and contribute to the development of renal phenotypes associated with immune-related abnormalities [30].

Based on the KEGG analysis, the main pathways identified include the MAPK signaling pathway, apoptosis, calcium signaling pathway, JAK-STAT signaling pathway, and TNF signaling pathway. Among these, the apoptosis pathway demonstrated significant prominence in the KEGG analysis. Furthermore, both STAT3 and apoptosis-related CASP3 proteins played central roles in the PPI network. Notably, the central target MAPK14 in the PPI network acted as the primary conduit for the MAPK signaling pathway, which is involved in regulating cell proliferation and apoptosis. These findings suggest that Danshenol C may promote apoptosis or inhibit the proliferation of peritoneal fibrotic cells and the formation of PF blood vessels through modulation of these target proteins. In addition, Danshenol C displayed a superior ability to regulate numerous cell cycle-related signaling pathways and inflammatory pathways, with particular significance observed in the TNF and MAPK signaling pathways. MAPK14, being central in the PPI network, also played a crucial role in mediating inflammatory responses associated with various diseases. Moreover, the MAPK pathway in the calcium signaling pathway was also implicated in aspects of cell cycle regulation [31]. Calcium ions, by modulating their movement within the joint, influenced nutrient supply, osmotic pressure, and cell apoptosis. By modulating these cell cycle and inflammatory pathways, Danshenol C holds the potential for cellular protection, peritoneal preservation, and anti-inflammatory effects [32, 33].

The objective of this study was multifaceted, encompassing several key findings and significant observations: ① Differential effects of glucose peritoneal dialysate on peritoneal mesothelial cells: Our investigation revealed that varying concentrations of glucose peritoneal dialysate exerted distinct effects on peritoneal mesothelial cells. Higher glucose concentrations correlated with increased fibrosis severity. Specifically, peritoneal dialysate containing 4.25% glucose induced fibrosis in a majority of normal HmRSV5 cells within 48 h, as evidenced by the gradual transition of cell morphology into a spindle shape. Additionally, the protein expression of vascular endothelial growth factor A (VEGF-A) increased, while the protein expression of the epithelial marker E-cadherin decreased, indicative of the occurrence of epithelial-mesenchymal transition (EMT). ② Danshenol C's cytotoxicity and safety: Our evaluation of the cell viability of HmRSV5 cells revealed that 48 h of intervention with different concentrations of Danshenol C alone did not significantly alter cell viability. This result underscores the lower cytotoxicity and higher safety profile of Danshenol C. ③ Reversal of PF by Danshenol C: Co-treatment of HmRSV5 cells with different concentrations of Danshenol C and peritoneal dialysate containing 4.25% glucose for 48 h demonstrated that 10 μM and 20 μM Danshenol C significantly increased cell viability. Moreover, Danshenol C reversed the cell morphology changes associated with fibrosis, leading to decreased VEGF-A protein expression and increased E-cadherin protein expression, indicative of its potential to reverse PF. ④ Identification of key targets for Danshenol C's therapeutic action: Real-time PCR analysis revealed that Danshenol-treated cells exhibited significant reductions in mRNA expressions of MAPK8 (JNK1), MAPK14 (P38), and STAT3, while the mRNA expression of CASP3 was significantly increased. These results strongly suggest that the treatment of PF with Danshenol C is closely associated with the modulation of these four critical targets.

Two intriguing observations emerged from our experimental findings. As previously mentioned, caspase 3 was associated with apoptosis. Both mRNA and protein expression increased after Danshenol C treatment, but we found no statistical difference between control group and experimental group by flow analysis of apoptosis. So there may be other effects for the caspase 3 increase caused byDanshenol C. Besides, the mRNA expression levels of MAPK14 showed a significant decrease. However, contrary to this, the protein expression levels increased upon co-culturing Danshenol C with high glucose peritoneal dialysate. Several potential explanations for this phenomenon are considered: ① The stability of mRNA changes: It is possible that certain proteins, like Nucleolin, may play a role in mRNA stability. Nucleolin has the capacity to bind to the transcription initiation factor (eIF3) complex, thereby regulating mRNA stability and translation efficiency of specific proteins (23). ② Danshenol C's effects on other mRNAs: Danshenol C may influence the expression of various other mRNAs, which in turn could impact the expression levels of MAPK14. ③ Inhibition of protein degradation: Danshenol C may inhibit protein degradation, leading to protein accumulation and subsequently impacting mRNA synthesis. ④ Fragment sites in STAT3 and P38: STAT3 and P38 proteins contain several fragment sites. While the expression of specific fragment sites may have increased in our experiment, other fragment sites might exhibit decreased expression. To gain a comprehensive understanding of the underlying mechanisms and how Danshenol C precisely regulates EMT (Epithelial-Mesenchymal Transition), our research team plans to employ advanced molecular interaction techniques, such as RIP (RNA Immunoprecipitation), CoIP (Co-immunoprecipitation), and double luciferase assay. These sophisticated methods will enable us to explore the intricate molecular interactions and pathways through which Danshenol C exerts its effects on EMT regulation. Through further investigations employing these molecular techniques, we anticipate unraveling the fascinating and complex molecular interactions influenced by Danshenol C. By shedding light on these underlying mechanisms, our research aims to contribute significant insights into the therapeutic potential of Danshenol C in the context of EMT regulation, further advancing the understanding of PF treatment.

## Conclusion

In summary, this study represents the first exploration of the potential of Danshenol C as a novel therapeutic compound for PF. We have made preliminary discoveries regarding the molecular network targets affected by Danshenol C in PF intervention. Through our investigation, we have identified key molecular targets, including STAT3, MAPK14, MAPK8, CASP3, and others, which are regulated by Danshenol C. This regulation leads to the activation of specific signaling pathways, such as MAPK, JAK-STAT signaling, and TNF signaling pathways, critical in the context of PF treatment.

These significant findings serve as a scientific foundation for the future clinical development of drugs related to Danshenol C and open up new possibilities for targeted therapeutic interventions in PF. It is worth noting that further studies are necessary to validate the protein molecular targets for docking in future investigations. By confirming and expanding upon our findings, we can gain deeper insights into the precise molecular interactions and mechanisms underlying Danshenol C's therapeutic effects. Overall, this research lays the groundwork for the potential clinical application of Danshenol C as a treatment option for PF. Further investigations and validation studies are warranted to fully unlock the therapeutic potential of Danshenol C and advance the field of PF treatment.

### Supplementary Information

**Additional file 1.**

## Data Availability

All data and results of the current study are available from the corresponding authors upon reasonable request. The manuscript data were obtained from the following public databases: YaTCM database (http://cadd.pharmacy.nankai.edu.cn/yatcm/help). SwissTargetPrediction database(http://www.swisstargetprediction.ch/). Uniprot (https://www.uniprot.org/). GEO DataSets(https://www.ncbi.nlm.nih.gov/gds). GeneCards (https://www.genecards.org/). PharmGKB (https://www.pharmgkb.org/). DrugBank (https://www.drugbank.ca/). OMIM (https://omim.org/). TDD (http://db.idrblab.net/ttd/). STRING database (https://string-db.org/cgi/input.pl). GO(http://geneontology.org/). KEGG(https://www.kegg.jp/). RSCBPDB database(https://www.rcsb.org/). PubChen database(https://pubchem.ncbi.nlm.nih.gov/).
