# Supplementary material for: Molecular mechanism of Danshenol C in reversing peritoneal fibrosis: novel network pharmacological analysis and biological validation
Source: BMC Complement Med Ther. 2023 Oct 13;23:361. doi: 10.1186/s12906-023-04170-x (PMC10571429; doi:10.1186/s12906-023-04170-x)
Supplement: Supplementary file 1 — Additional file 1. [file 12906_2023_4170_MOESM1_ESM.pdf]

GAPDH

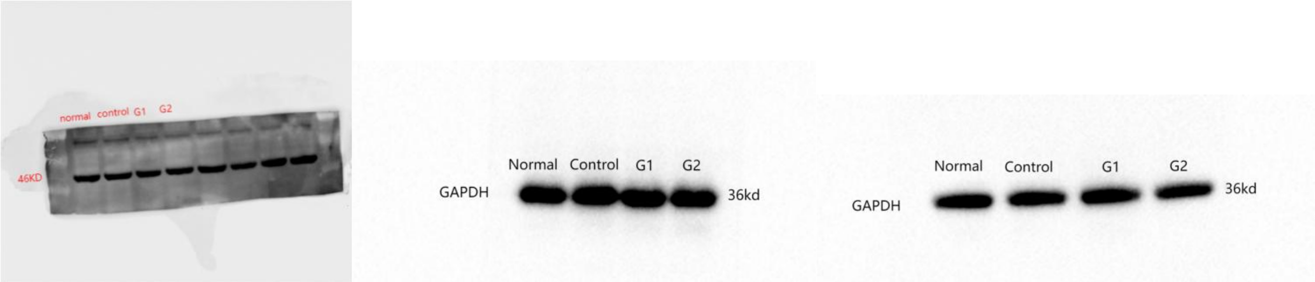

STAT3(T721)

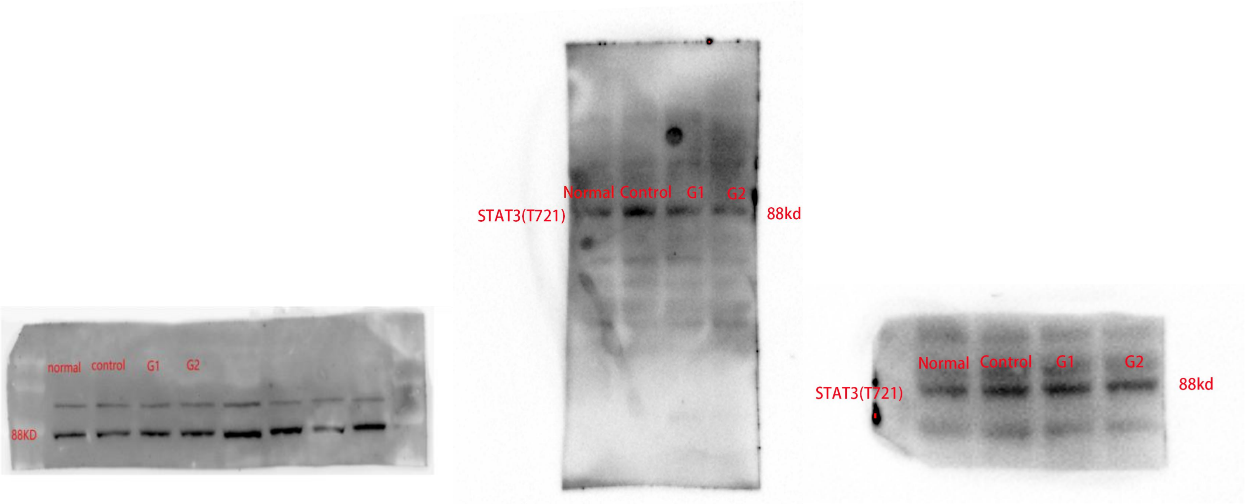

STAT3(S727)

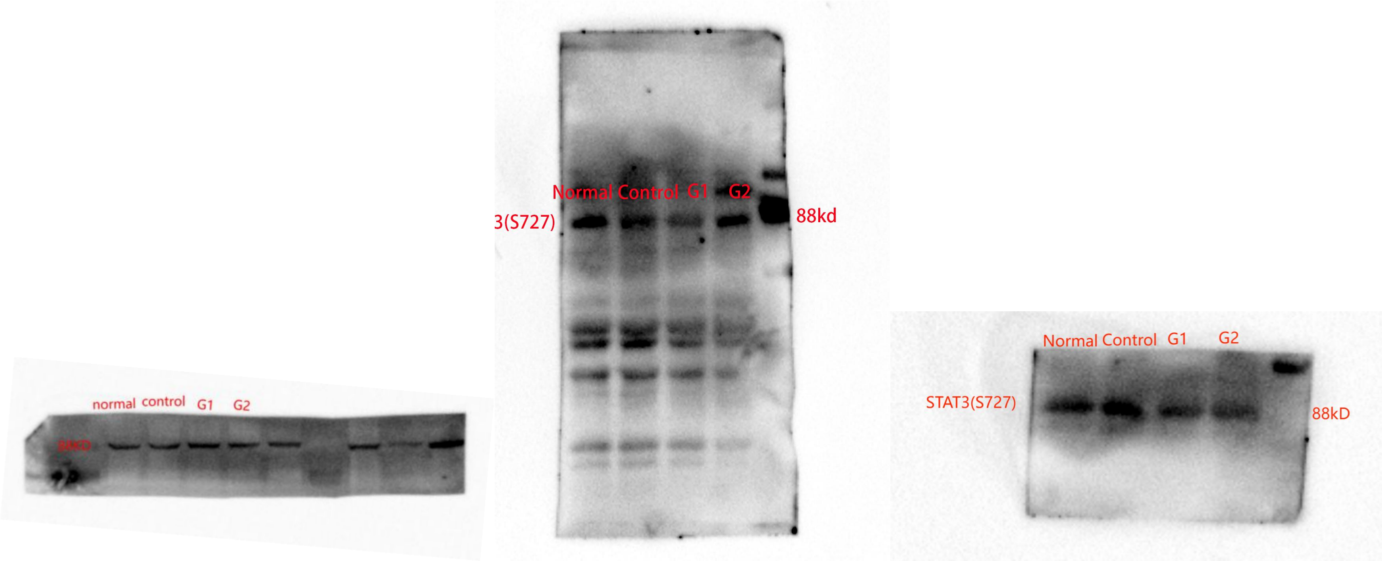

Since little spacing above or below the target bands, the blots were cut prior to hybridisation with antibodies during blotting.

STAT3(Y705)

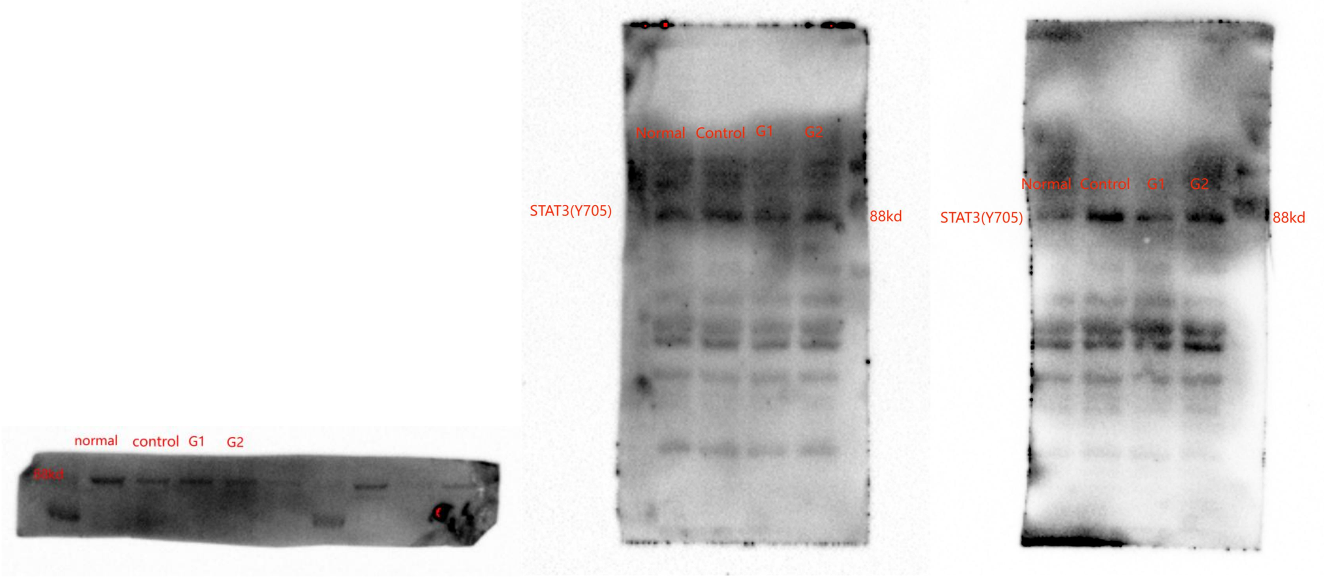

P38(H174)

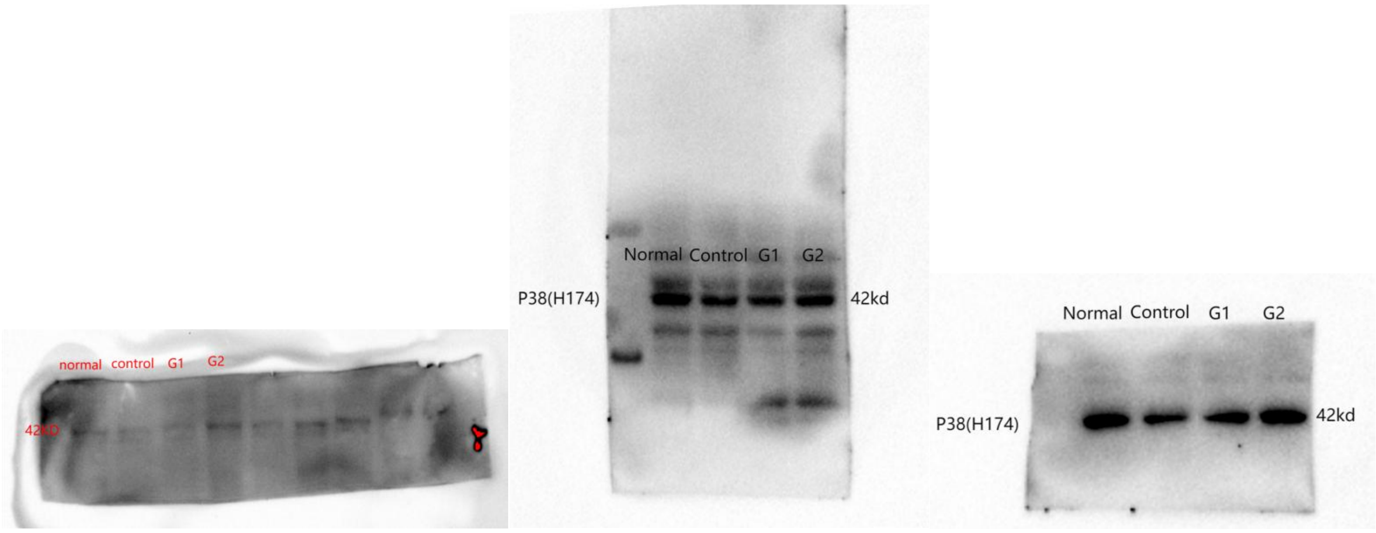

p-P38(T180/182)

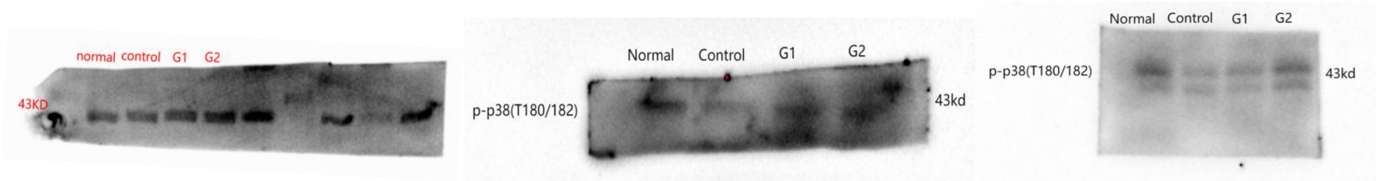

Since little spacing above or below the target bands, the blots were cut prior to hybridisation with antibodies during blotting.

Caspase3

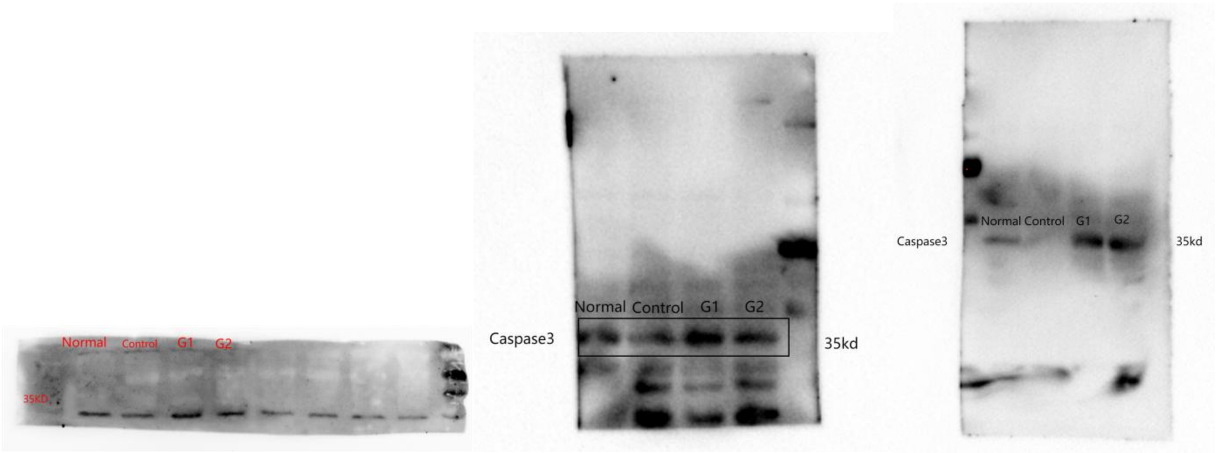

JNK1

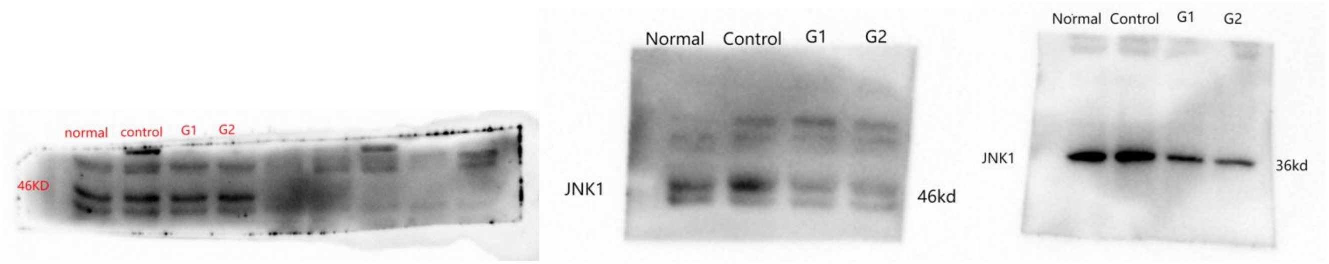

GAPDH(vegf,e-cad)

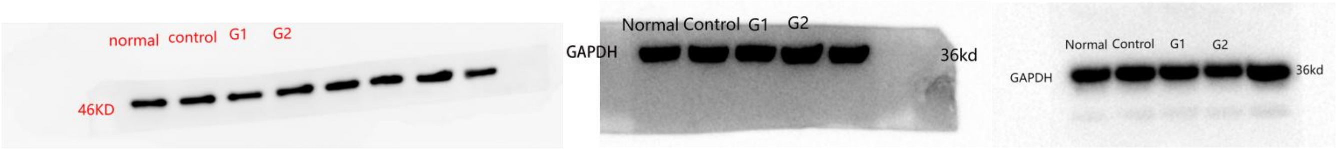

VEGF-A

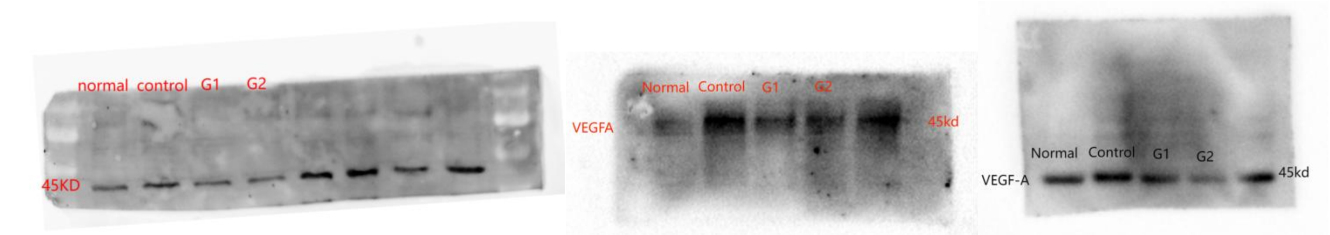

E-Cadherin

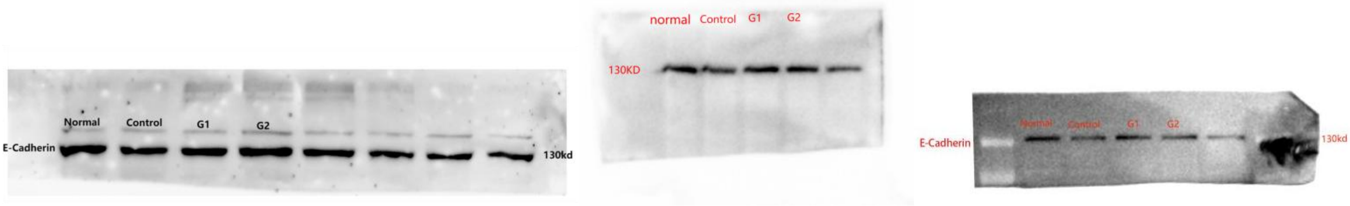

Since little spacing above or below the target bands, the blots were cut prior to hybridisation with antibodies during blotting.
